# Targeted Anthocyanin Enrichment of Cranberry Juice by Electrodialysis with Filtration Membranes: Impact of Filtration Membrane Physicochemical Properties and Predictive Statistical Models

**DOI:** 10.3390/membranes14050111

**Published:** 2024-05-14

**Authors:** Eva Revellat, Laurent Bazinet

**Affiliations:** 1 Food Sciences Department, Institute of Nutrition and Functional Foods (INAF), Université Laval, Québec, QC G1V OA6, Canada; eva.revellat.1@ulaval.ca; 2 Laboratoire de Transformation Alimentaire et Procédés ElectroMembranaires (LTAPEM, Laboratory of Food Processing and ElectroMembrane Processes), Université Laval, Québec, QC G1V OA6, Canada

**Keywords:** cranberry, electrodialysis, filtration membrane, anthocyanins, membrane physicochemical properties

## Abstract

To optimize cranberry juice enrichment, correlation between physicochemical properties of filtration membranes (FM) and anthocyanin migration was investigated during electrodialysis with filtration membranes (EDFM) using redundancy (RDA) and multivariate regression (MRGA) analyses. Six polyether sulfone (PES) and polyvinylidene fluoride (PVDF) membranes with molecular weight cut-offs between 150 and 500 kDa, commercially available at large scale, were characterized in terms of nine physicochemical characteristics and used for EDFM. The highest migration of total anthocyanin was obtained with PVDF 250 kDa, with a global migration rate of 3.5 ± 0.4 g/m^2^·h. RDA showed that two FM properties (mesopore porosity and hydrophilic porosity) were significantly negatively correlated to the anthocyanin’s migration and explained 67.4% of their total variation in migration. Predictive MRGA models were also developed for each anthocyanin based on these significant FM properties. A combination of intermolecular interactions may lead to binding in a cooperative and synergistic mode and hinder the anthocyanin migration.

## 1. Introduction

Rise of metabolic, cardiovascular diseases induced by higher consumption of processed and hypercaloric foods encourage consumers to pursue a diet with food containing natural bioactive compounds [1,2]. Studies on polyphenolic compounds have highlighted an important number of beneficial effects on human health [3,4,5,6]. However, the polyphenol family is composed of a large range of molecules, and anthocyanins are a class of compounds among them. Anthocyanins formed of an aglycon (anthocyanidins) and a sugar moiety are used as food colorant but also as nutraceutical ingredients [7]. The abundance of anthocyanins in cranberry berries and in its transformed form cranberry juices, which represent 60% of the cranberry production [8], makes them the ideal candidate for enrichment to enhance its heath benefit and consumption. Hence, health-promoting impacts attributed to anthocyanins have been extensively studied and researchers continue to discover new health benefits. One of the main focus was done on cardiovascular and neurodegenerative diseases [7]. More recently discoveries have suggested their impacts on the modulation of the gut microbiota [9,10,11].

Fractionation and purification of polyphenols (often present in food matrices) is complex, since polyphenols differ in terms of structure, composition and physicochemical property, forcing the selection of appropriate separation technologies. A wide variety of physicochemical methods have been evaluated for their ability to remove phenolic compounds. Hence, current extraction methods include microwave extraction [12], organic solvent extraction [13], supercritical fluid extraction [14], ultrasonic assisted extraction [15], adsorption on resins [16] or extraction using enzymes [17]. These processes have many disadvantages, e.g., organic solvent extraction is inefficient and can be dangerous due to the toxicity of the solvents [18]. Some phenolic compounds can also be degraded under the high temperature conditions generated by microwave extraction. The use of a co-solvent is necessary in the case of supercritical fluid extraction (SFE) to increase the solubility of certain compounds and increase the selectivity of the extraction [19]. Other methods, which are widely used, are membrane filtration processes. Indeed, these technologies allow fractionation according to molecular size (through pressure-driven processes), charge, or both (electrical processes) in the case of a new electro membrane approach [20]. Electrodialysis with filtration membrane (EDFM) is appraised as a rising process for the separation of bioactive molecules from natural sources [20]. An initial study [21], employing a filtration membrane in an EDFM cell, has produced a polyphenol enriched cranberry juice fraction from a bigger volume of cranberry juice. Applied electric field drives selective migration of polyphenol compounds throughout the FM to the cathode side, due to the positive charge of these molecules. After 4 h, an enrichment of 52.9% and 34.8% was obtained in anthocyanins and proanthocyanins, respectively. However, the raw juice polyphenol concentration was diminished through the constant recirculation of the raw juice. In terms of an industrial integrated enrichment process, it would therefore be necessary to optimize this enrichment by selecting the parameters and the physicochemical properties of the FM most critical to the migration of polyphenols.

In this context, the objectives of this study were (1) to characterize membrane properties, (2) to determine the effect of MWCO and membrane material on the migration of polyphenols (anthocyanins, proanthocyanidins (PAC) and organic acids) during EDFM, (3) to complete redundancy analysis to identify the most significant FM properties that impact global, individual anthocyanin migrations and organic acid migration, and (4) to implement multivariate regression analysis to create predictive models for the global, individual anthocyanin migrations and organic acid migration based on the significant FM properties.

## 2. Materials and Methods

### 2.1. Materials

#### 2.1.1. Cranberry Juice

**Figure 1:**
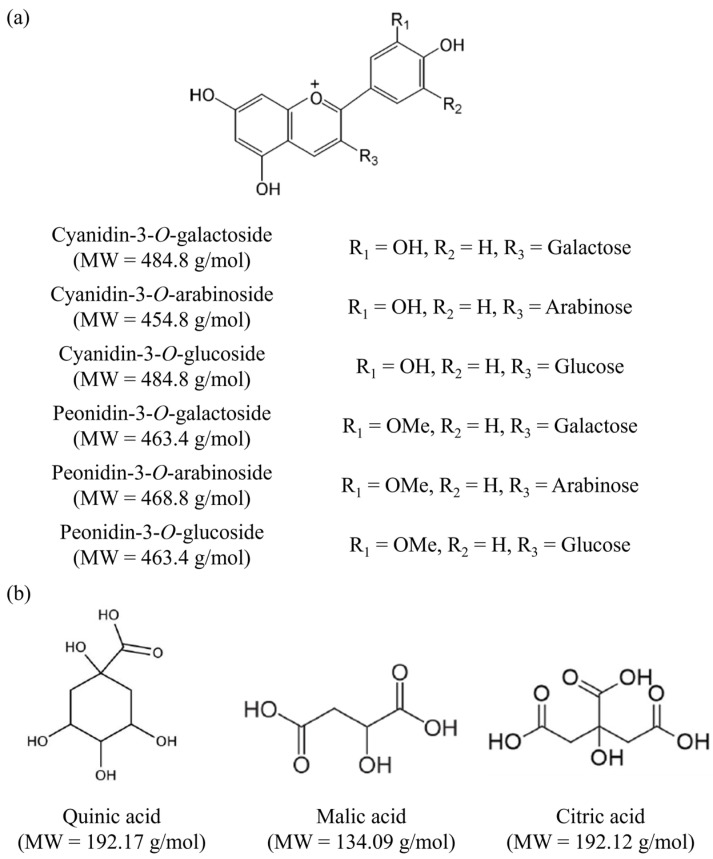
Chemical structures and molecular weights (MW) of principal (**a**) anthocyanins and (**b**) organic acids identified in cranberry juice (Figure adapted from [23]).

The same lot of 8° brix cranberry juice manufactured by Fruit d’Or (Plessisville, Québec, Canada) was used for all EDFM treatments and characterized in terms of physicochemical properties (Table 1). The juice was thawed before experiments and was stored at 4 °C. Total anthocyanin concentration reached 197 mg/L in our study, which is in accordance with values reported in the literature. Indeed, anthocyanin concentrations in cranberry juice can vary between 27 mg/L and 199.4 mg/L according to the works of Faucher et al. [22] and Blumberg et al. [8]. Structures of anthocyanins and organic acids are detailed in Figure 1. 

#### 2.1.2. Membranes and EDFM Configuration

A microflow type cell (ElectroCell AB, Karlskoga, Sweden) with an effective area of 10 cm^2^ was installed with Neosepta cation-exchange membranes (CMX-Fg), and anion-exchange membranes (AMX-Fg), both purchased from Ameridia (Napa, CA, USA), and one filtration membrane (FM). Six filtration membranes were selected for this study (Table 2). Selection of the FMs was based on their constitutive material and cut-offs as well as their commercial availability at large scale and FDA grade. Commercially available membranes were selected in the event of scaling up. Thus, the MWCO selected were not identical for the two types of membranes. 

**Figure 2:**
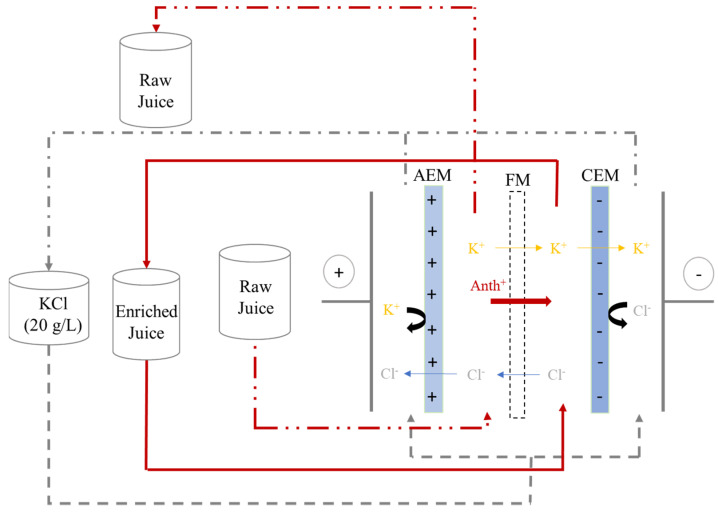
EDFM System Configuration (AEM = Anion exchange membrane, FM = Filtration membrane, CEM = Cation exchange membrane).

In this configuration, ion exchange membranes prevent polyphenol migration from juice compartments and thus avoid the blending of juice with electrode rinsing solutions. The cell consisted of three closed loops: one is by-passed and connected to an external reservoir to avoid recirculation of the solution; the two others are linked to their respective reservoir for solution recirculation during treatment. Three centrifugal pumps and three flow meters allow a controlled circulation of the solution (Figure 2). The cathode was a 316 stainless steel electrode and the anode a dimensionally stable electrode (DSA). Anode/cathode voltage difference was delivered by an electric power supply (BK Precision 9110, Yorba Linda, CA, USA).

### 2.2. Protocol: Membrane Property Characterization and EDFM Experiments

Before any EDFM treatment, physicochemical characteristics of all six FMs were analyzed: conductivity, roughness, thickness, surface potential, contact angle, porosity, nature of pores and pore size distribution.

A treatment duration of 180 min was selected, according to [24], to enhance the enrichment in anthocyanins of the enriched juice. A 9 L volume of raw juice was circulated twice while a volume of enriched juice of 300 mL was recirculated. The 9 L of juice were added gradually to the system to ensure an equivalent volume on both sided of the FM. Thus, according to these authors [24] after two loops, the juice characteristics are not jeopardized. In the electrode rinsing compartment, a 20 g/L KCl solution was circulated. Cranberry juice flow rates were 100 mL/min while the flow rate of the KCl solution was 400 mL/min. Treatments were performed in batch process at room temperature. For a distance between the two electrodes of 2 cm, the applied voltage was 30 V. At 0, 60, 120 and 180 min, samples of raw juice and enriched juices were aliquoted to characterize the evolution of juice color, degree brix, anthocyanin concentration, proanthocyanidin concentration and organic acid concentration. All along the EDFM, the conductivity, pH, intensity, and voltage were measured. Three replicates of each treatment were performed. 

### 2.3. Analyses

#### 2.3.1. Physicochemical Properties of FM

The membranes’ physicochemical properties were characterized before EDFM treatment. For each type of analysis, three different pieces of the same membrane were analyzed.

Zeta potential

An electrokinetic analyzer (SurPASS, Anton Paar, Graz, Austria) was used to measure the zeta potential, equipped with a clamping cell. The measurement’s principle was described by [25]. For each membrane, the streaming current in a 1 mM KCl solution was analysed in a pH range of 2 to 7.

Roughness

To measure average roughness (Ra) and average peak-to-valley profile (Rz) and determine a three-dimensional topography picture of the membrane surface, a micro-stylus profilometer (DektakXT, Bruker Nano Surfaces, Tucson, AZ, USA) was operated [25]. Once the 5 µm tip of the stylus had encountered the membrane and ran over in a hill and valley modes and a vertical measurement range of 524 µm, 50 scans were obtained. Each scan lasts 5 sec and covers 1000 µm. All the data were analyzed thanks to Vision64 software (Version 5.51, Bruker Nano Surfaces, Tucson, AZ, USA).

Thickness

Membrane thickness was measured with a Marathon Electronic Digital Micrometer (Marathon Watch Company Ltd., Richmond Hill, ON, Canada). A mean thickness of six measurements of each membrane was calculated [25].

Contact angle

The contact angle value was determined with a goniometer Theta OneAttension (Version 2.4, Biolin Scientific, Linthicum Heights, MD, USA). As detailed by [25], a high-resolution camera captured a drop of 2 μL of distilled water on the membrane, which is placed and stabilized on a microscope slide on a flat support. Angle range of measurement was from 0° to 180°. This was carried out in triplicate for the same piece of membrane and with triplicate measurement. All contact angle data were analyzed through OneAttension software (Version 2.4, Biolin Scientific, Espoo, Finland).

Conductivity

The conductivity was measured at three positions on the membrane surface with a specially designed clip from the Laboratoire des Matériaux Echangeurs d’Ions (Université Paris XII, Créteil, France) as previously reported by [25].

Porosity, nature of pores and pore size distribution (PSD)

An automated standard porosimeter was used (Automated Standard Porosimeter 3.2, MPM and P Research Inc., Newmarket, ON, Canada) to determine different membrane porosity parameters. The method draws on the laws of capillary equilibrium; in other word, values of capillary pressure of two porous bodies filled with wetting liquid in contact with each other in state of capillary equilibrium are equals. Knowing distribution size of a standard, then distribution size of the sample can be resolved by calculating the distribution of the liquid between all the bodies [25]. Discs of 23 mm were placed on the sample holder with a drying device. Automated arms allowed collection, dismantling and transfer to the balance of the sample and standard pile. Standards and membranes were vacuumed and impregnated in octane or water. Determination of total porosity and hydrophilic porosity was performed using octane (with contact angle equal to 0) and water as operating liquid. For both liquids, three discs were tested [25]. Data were processed through POROVOZ software (Version 09, MPM and P Research Inc., Toronto, ON, Canada) allowing the generation of a graph of differential distribution (dV/d (log r)) of pore volume (V) vs pore radius (log r). 

#### 2.3.2. Juice Characterization

pH

During the treatment, using a pH-meter (Models Orion Star A221, Thermo Scientific, MA, USA), pH of both raw and enriched juices was measured.

Conductivity

At 0, 5, 10, 15, 60, 120 and 180 min, enriched and raw juice conductivity were measured with a YSI conductivity meter (Model 3100) using a YSI immersion probe (Model 3252, cell constant K = 1 cm^−1^; Yellow Springs Instruments Co., Yellow Springs, OH, USA), as previously described by [24].

Degree Brix

Degree brix of both raw and enriched juice were determined thanks to a digital hand-held refractometer PAL-1 (ATAGO, Tokyo, Japan).

Anthocyanin content and migration rate

Individual anthocyanin contents of the raw and enriched juices were analyzed by HPLC. Prior to the injection of 20 μL of samples in the system, samples were diluted (dilution factor = 1.25) in a 0.5% TFA methanol solution. This acidic solvent made anthocyanins color stand out. Then, samples were filtered on a 0.45 μm nylon filter. The column used (at room temperature) for the separation was a ZORBAX SB-C18 column (Agilent, 5 µm, 4.6 × 250 mm, Santa Clara, CA, USA) combined with a DAD detector with a detection wavelength of 520 nm. The cyanidin-3-glucoside (MW = 449.2 g·mol^−1^ and ε = 26 900 L·mol^−1^·cm^−1^) was used as standard. The elution time was 1 mL/min. The two mobile phases used were a water/formic acid (95%/5%) and 100% methanol solutions [26]. 

For all membranes tested, global and individual anthocyanin migration rates (g/m^2^·h) were calculated by dividing the total quantity of anthocyanins (g) migrated toward the cathode at the end of the EDFM by the effective surface area of FM (m^2^) and the duration of the EDFM process (h).

(1) $$\mathrm{Anthocyanin}\;\mathrm{Migration}\;\mathrm{rate}\;\left(\frac{g}{m^{2}\cdot h}\right)=\frac{\left(C_{f}-C_{0}\right)\left(\frac{g}{L}\right)\times \;\mathrm{Enriched}\;\mathrm{Juice}\;\mathrm{volume}\;\left(L\right)}{\mathrm{area}\;\left(m^{2}\right)\times \mathrm{Time}\;\left(h\right)}$$

With C_f_ and C_0_, the final and initial concentrations in anthocyanins of the enriched juice.

The enriched juice’s enrichment yields were determined using the following equations:

(2) $$Y_{enrichment}\left(\%\right)=\frac{\left(C_{f}-C_{0}\right)\left(\frac{g}{L}\right)}{C_{0}\left(\frac{g}{L}\right)}\times 100$$

Organic acid content and migration rate

A HPLC system provided with a UV detector (detection wavelength of 214 nm) and equipped with a Synergi Hydro-RP 80 A column (250 mm × 4.6 mm, Phenomex, Torrance, CA, USA) was used to determine the organic acid content [24]. The flow rate was 0.8 mL/min and the mobile phase was made of a 0.2 M KH_2_P0_4_ solution at pH 2.4. The volume of sample injected in the column was 10 μL. Malic, citric and quinic acid standards (Sigma Aldrich, Saint-Louis, MO, USA) were used to determine calibration curves and retention times and thus, the organic acid concentrations. 

For all membranes tested, global and individual acid organic migration rates (g/m^2^·h) were calculated by dividing the total quantity of acid organic (g) migrated toward the anode at the end of the EDFM by the effective surface area of FM (m^2^) and the duration of the EDFM process (h).

(3) $$\mathrm{Organic}\;\mathrm{acid}\;\mathrm{migration}\;\mathrm{rate}\;\left(\frac{g}{m^{2}\cdot h}\right)=\frac{\left(C_{f}-C_{0}\right)\left(\frac{g}{L}\right)\times \mathrm{Enriched}\;\mathrm{Juice}\;\mathrm{volume}\;\left(L\right)}{\mathrm{area}\;\left(m^{2}\right)\times \mathrm{Time}\;\left(h\right)}$$

With C_f_ and C_0_, the final and initial concentrations in organic acids of the enriched juice.

Proanthocyanidin content

To determine proanthocyanidin concentrations, an Agilent 1260 Series HPLC system (Agilent Technologies, USA) was used. Prior to HPLC analysis, samples were diluted (dilution factor of 200/59) in 0.71% acetic acid acetone solution. Nomura chemical Develosil 100 Diol-5 column (4.6 × 250, Phenomenex, Torrance, CA, USA) at 35 °C and a fluorescence detector (Agilent Technologies, Palo Alto, CA, USA) with emission and excitation wavelengths equal respectively to 321 and 230 nm were used. An epicatechin standard curve was used to estimate all PACs with different degrees of polymerisation. Separation of PACs, based on their polymerization degree was performed with solvent A, acetonitrile/acetic acid (98%/2%) and solvent B, methanol/water/acetic acid (95%/3%/2%) for elution at 0.8 mL/min [27].

Juice color

A color representation system with three parameters L* (lightness), a* (color intensity differing from red to green) and b* (color intensity differing from yellow to blue) allowed the determination of the juice color. A Minolta chromameter (Model Minolta meter CR-300, Konica Minolta Inc., Mississauga, ON, Canada) was used to measure the color of the juices [24]. 

### 2.4. Statistical Analyses

#### 2.4.1. ANOVA 

Each membrane was tested in the EDFM system in triplicate and each repetition were randomly split in three blocks. The mean value and standard deviation were calculated from the three repetitions. Data obtained for FM properties, global anthocyanin and individual anthocyanin migrations were treated with a one-way ANOVA and using a Tukey test; significative differences between FMs were defined at a probability level of *p* < 0.05. 

#### 2.4.2. Redundancy Analyses and Predictive Statistical Models

To reveal a linear correlation between explanatory and response variables, multivariate statistical methods (RDA and multivariate regression analysis) were employed. RDAs allowed to understand the influence of FM physicochemical properties (which correspond to explanatory variables) on anthocyanin and organic acid migrations and selectivities (response variables) during EDFM. Thus, RDA and multivariate regression analysis were used to determine the link between the nine physicochemical properties (thickness, surface roughness, volumetric porosity, conductivity, zeta potential, contact angle, hydrophilic porosity and mesopore porosity) of the six membranes (see Table 2) and anthocyanin or organic acid migration. In the EDFM cell, the use of the characterized membranes was impossible due to some destructive analysis methods. So, for statistical purpose and better accuracy, seven groups of characteristics that were measured independently of each other were defined, since there was no link in the order of entry of the repetitions of certain characteristics measured: (1) thickness, (2) conductivity, (3) zeta potential, (4) contact angle, (5) Ra and Rz, (6) hydrophilic, total and mesopore porosities, (7) anthocyanins and organic acid migration. To consider the absence of link between these seven groups of variables, one thousand data sets were created with random permutations of these seven groups of explanatory and response variables [28]. This makes it possible to perform the statistical analyzes separately with 1000 possible input orders of the characteristics measured. Results of these 1000 statistical analyzes were then combined to present global results considering the variability within and between the 1000 entry orders. The main reason for using 1000 random permutations of variable group values is that conclusions of statistical analyzes may greatly vary depending on the order of data entry. Then, collinearity tests between the explanatory variables of these 1000 data sets were carried out [28,29,30]. Then, to assess bivariate association (whether linear or not), the combined Spearman correlation considering the 1000 generated random input orders was calculated between the independent variables and the dependent variables [31]. A selection of variables with the “ordistep” (an automatic stepwise model) function of the R software was carried out for each of the 1000 data sets as part of a redundancy analysis. Features retained in at least 95% of the 1000 datasets were then included in a final redundancy analysis, thus significant characteristics were selected with a 5% threshold, as previously performed by [25]. Finally, linear regression models were carried out with the variables retained by the redundancy analyses, considering the estimation of the parameters of the 1000 data sets.

## 3. Results

### 3.1. Physicochemical Properties of Filtration Membranes

Amongst the six membranes described (Table 2), the most conductive membrane appeared to be the PVDF 250 kDa (12.8 ± 0.2 mS/cm), whereas the PES 150 kDa had the lowest conductivity value, and the four other membranes showed similar conductivities with an average and intermediate value of 11.1 ± 1.2 mS/cm (Table 3). At pH of the juice of 2.5, only one membrane revealed a positive surface charge: the PVDF 150 kDa (3.4 ± 1.2 mV). The PVDF 250 kDa followed with a surface charge just below 0 mV. The four other membranes displayed negative charges. Among them, PES 150 kDa and PES 300 kDa had the highest negative zeta potentials followed by the PES 200 kDa. With contact angle value going from 57° to 78°, the FMs studied had a hydrophilic to slightly hydrophilic surface: PVDF 150 kDa had the highest contact angle value whereas the PES 300 kDa displayed the lowest. Regarding surface topography, roughness Ra of all membranes studied was similar, with a mean value of 1.21 ± 0.22 μm. In addition, all FMs were found to have close total volumetric porosity value, with an average of 0.64 ± 0.02 cm^3^/cm^3^. Nevertheless, PES 200 kDa and PES 150 kDa displayed a higher Rz meaning both membranes have a higher average roughness. Membranes were dissimilar in pore size distribution. The PES 200 kDa exhibited the lowest percentage of macropores (79 ± 6%) compared to the five other membranes presenting similar values (91 ± 2%). However, the PES 200 kDa had the highest volumetric porosity of mesopores (0.111 ± 0.030 cm^3^/cm^3^) whilst the PES 150 kDa exhibited the smallest value (0.032 ± 0.003 cm^3^/cm^3^). As regards the nature of pores, only the PVDF 500 kDa had less than 50% of hydrophilic pores followed by the PVDF 250 kDa and then PVDF 150 kDa. Among all the membranes, PES membranes exhibited the highest percentage of hydrophilic pores. The data brought forward properties such as thickness, pore size distribution, conductivity, contact angle, percentage of hydrophilic porosity, surface charge and roughness Rz as different, either according to the membrane materials, their MWCO or both. However, roughness Ra and total volumetric porosity are similar for all membrane materials and MWCOs used.

### 3.2. Physicochemical Parameters of Cranberry Juices during EDFM

The conductivity, pH, color, and degree Brix of juices was assessed for all FM tested. No significant change appeared during the treatment whatever the type of FM employed in the EDFM. These results are detailed in the Supplementary Material Section (Figures S1 and S2 and Tables S1 and S2). 

### 3.3. Anthocyanin, PAC and Organic Acid Migrations

#### 3.3.1. Anthocyanin Migration 

**Figure 3:**
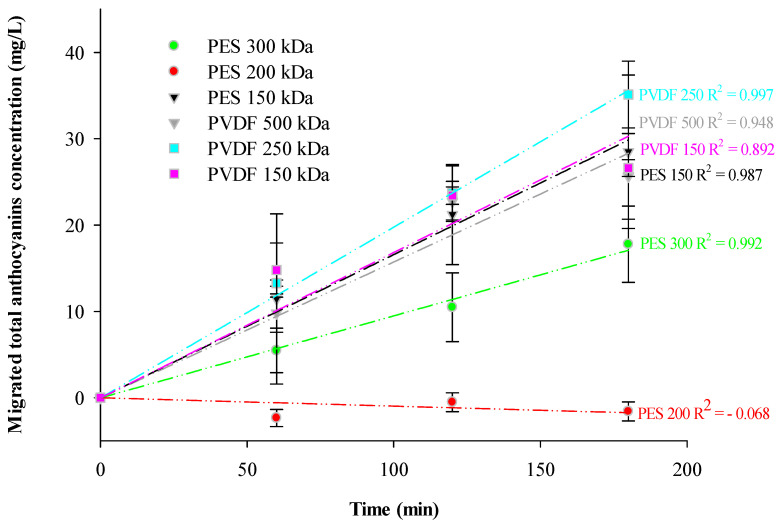
Total migrated anthocyanins during EDFM treatments for each tested membrane.

Data suggested that membrane materials and MWCOs had an impact on anthocyanin migration to the 300 mL compartment with significative difference in terms of anthocyanin migration rate between membranes (*p* < 0.0001) (Figure 3). With a global anthocyanin migration rate of 3.5 ± 0.4 g/m^2^·h, the highest migration of total anthocyanin was obtained with PVDF 250 kDa; this migration corresponds to a juice’s enrichment of 21.5%. Comparable migration rates were achieved for PES 150 kDa corresponding to an average global anthocyanins migration rate of 2.85 ± 0.39 g/m^2^·h. The following migration rates were obtained for PVDF 150 kDa and PVDF 500 kDa, with comparable migration rates of 2.66 ± 0.89 g/m^2^·hand 2.56 ± 0.50 g/m^2^·h, respectively. A lower migration rate of 1.78 ± 0.44 g/m^2^·hwas obtained with PES 300 kDa, significatively different from that for PVDF 250 kDa. These migration rates corresponded to a linear migration of anthocyanins for all membranes (except for the PES 200 kDa) as demonstrated by the R^2^ calculated (Figure 3). Indeed, no anthocyanin migration rate was observed with PES 200 kDa (−0.158 ± 0.11 g/m^2^·h). For the raw juice compartment, no impoverishment has been noticed for all membranes tested (result not shown). The analysis of variance did not show any significative (*p* = 0.4722) total anthocyanin concentration difference, with an average concentration of 197.0 ± 4.5 mg/L for the initial raw juice and an average concentration of 196.5 ± 2.9 mg/L for the final raw juice.

Data suggested a synergic impact of the MWCO and the membrane material on the global anthocyanin’s migration. A previous study [32] has reported weak enrichments, principally due to the low cut-offs of membranes, below 150 kDa. In the present study, the high cut-off selection removed this constraint and allowed a deeper understanding of the surface interaction and the impact of their constitutive material. Indeed, it has already been reported that a high retention rate of anthocyanins was not due to steric hindrance but to membrane interaction during pressure-driven membrane processes [33]. Hence, selective enrichment of cranberry juice in anthocyanins using a EDFM process without significant decrease of these molecules in the raw juice was possible. Those results confirmed previous findings [24]; however, the enrichment value achieved here after three hours of treatment is lower than expected compared to previous enrichment values obtained for cranberry juices [24,32]. But anionic and cationic membranes as well as the FM used in these previous studies were different from those employed here.

**Figure 4:**
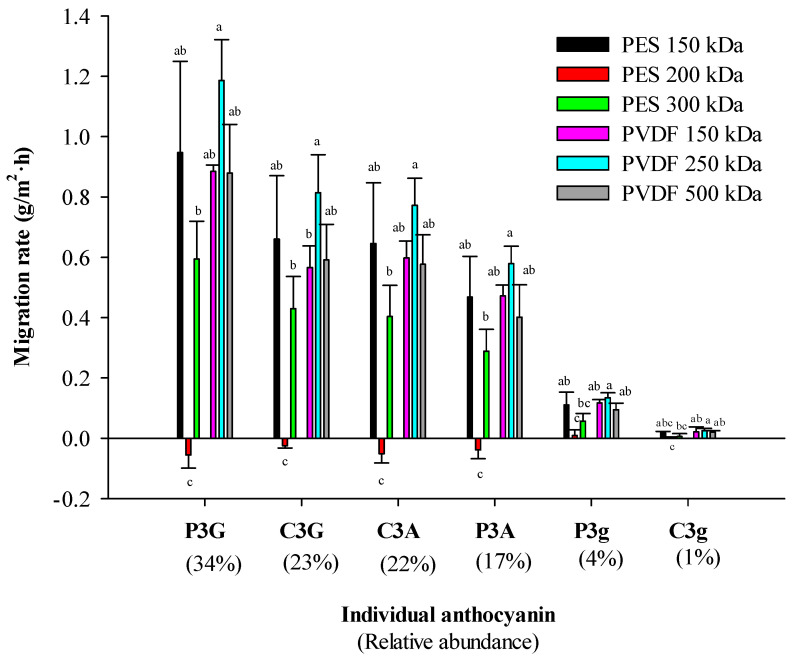
Individual anthocyanin migration rates for all membranes tested (P3G = Peodinin-3-Galactoside, C3G = Cyanidin-3-Galactoside, C3A = Cyanidin-3-Arabinoside, P3A = Peodinin-3-Arabinoside, P3g = Peodinin-3-glucoside and C3g = Cyanidin-3-glucoside).

Individual anthocyanin migration rates are presented in Figure 4 for PES and PVDF membranes. For all membrane tested, except PES 200 kDa, the results revealed that the highest migration rate of individual anthocyanins was correlated to the most abundant individual anthocyanins. Indeed, a similar individual anthocyanin ascending order was obtained for relative abundance and migration rate: peodinin-3-Galatoside (P3G) (34% of the total anthocyanins in cranberry juice), cyanidin-3-Galactoside (C3G) (23%), cyanidin-3-Arabinoside (C3A) (22%), peodinin-3-Arabinoside (P3A) (17%), peodinin-3-Glucoside (P3g) (4%) and cyanidin-3-Glucoside (C3g) (1%). However, according to the membranes used, migration rates of individual anthocyanin were significatively different. Comprehensively, the highest migration rate and selectivity were obtained with the PVDF 250 kDa. PVDF 500, PES 150 kDa and PVDF 150 kDa performed with comparable migration rates for each anthocyanin. However, PVDF 150 kDa seemed to have a better selectivity than PES 150 kDa and PVDF 500 kDa with a lower migration rate for C3G. Finally, PES 200 kDa gave the lowest migration rate and selectivity.

#### 3.3.2. PAC Migration

PAC concentrations in both compartments for all the FMs were determined by HPLC. Data suggest that membrane materials and MWCOs in the EDFM conditions tested did not allow the migration of PACs to the 300 mL compartment, whatever the membranes tested (*p* = 0.9025). For additional information on PAC migration, refer to the Supplementary Material Section (Figure S3).

#### 3.3.3. Organic Acid Migration

**Figure 5:**
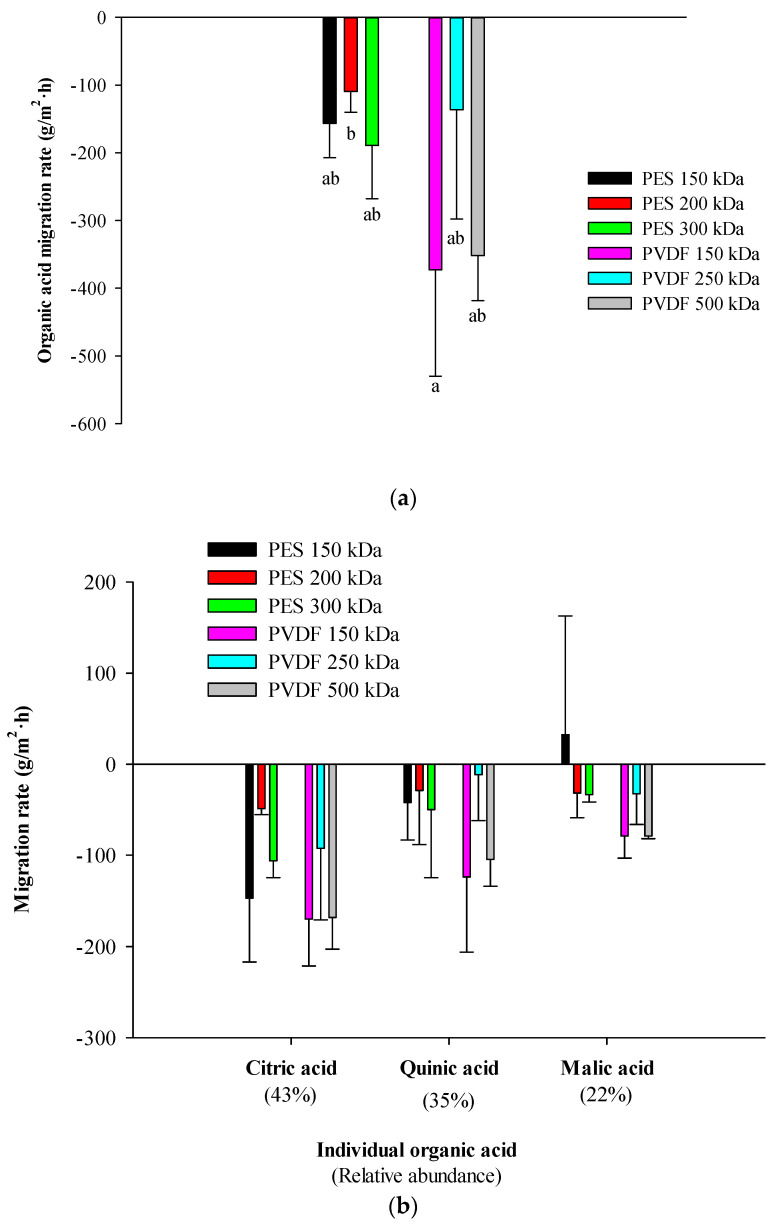
(**a**) Global and (**b**) individual organic acids’ migration rates during EDFM for each membrane tested.

Statistical analyses revealed that membrane materials and MWCOs had an impact on organic acid migration from the 300 mL compartment to the 9 L compartment with significant differences in terms of global organic acid migration rate between membranes tested in this study (*p* < 0.05) (Figure 5a). At the juice’s pH, few of the organic acids are in their negatively charged mono-ionized forms. Organic acid migration rate values were negative to show the opposite migration of organic from the 300 mL compartment to the 9 L compartment compared to anthocyanins. PES 200 kDa and PVDF 150 kDa are significatively different in terms of global organic acid migration: the highest global acid organic migration rate was achieved for PVDF 150 kDa (−372.80 ± 157.13 g/m^2^·h) followed by PVDF 500 kDa (−351.67 ± 66.66 g/m^2^·h) and the lowest was achieved for PES 200 kDa (−109.23 ± 30.97 g/m^2^·h). For all membranes tested, the global organic acid concentration in the 9 L compartment did not significatively increase due to the high volume of raw juice. Indeed, the initial total organic acid concentration was 35 588 ± 767 mg/L, and the final organic acid concentration was 35 513 ± 567 mg/L. 

Migration rates of the main cranberry juice individual organic acids (citric, malic and quinic acids) were comparable between EDFM treatments and for all FMs used (Figure 5b). (*p* > 0.05), but a global tendency of higher migration rate for the PVDF 150 kDa and PVDF 500 kDa was observed for each individual organic acid. As for individual anthocyanins, the highest migration rate was also obtained for the most abundant organic acid: migration of citric acid (−122 ± 48 g/m^2^·h) represented 58% of the total migration and was the most abundant acid in the juice (43%). The same applied for quinic acid (−58 ± 46 g/m^2^·h) and malic acid (−30 ± 34 g/m^2^·h), with 28% and 14% of the total migration and representing 35% and 22% of the total organic acids, respectively, in the raw juice. Such a selective migration of organic acid has already been reported but only during deacidification of cranberry juice and through the anion-exchange membrane [34].

### 3.4. Redundancy Analyses and Predictive Statistical Models 

**Figure 6:**
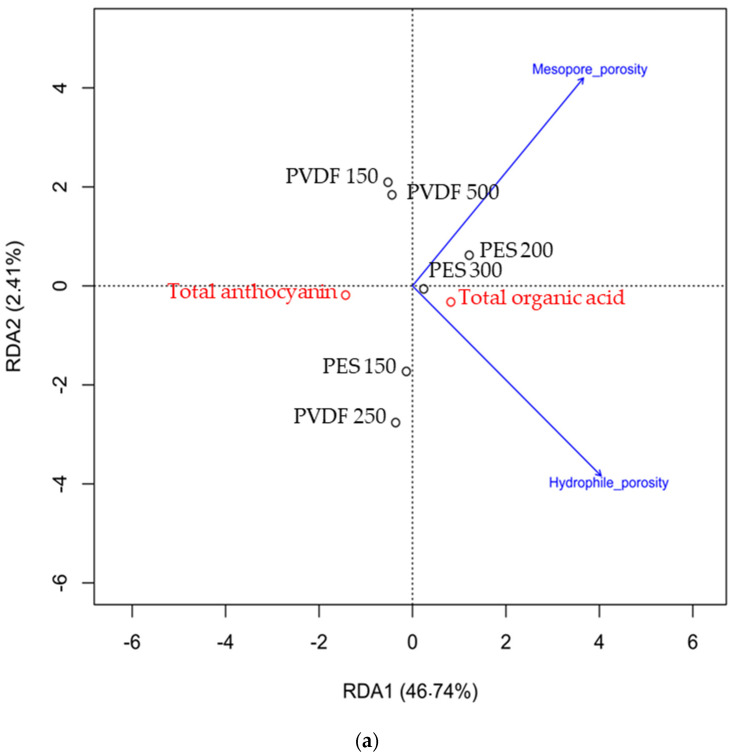

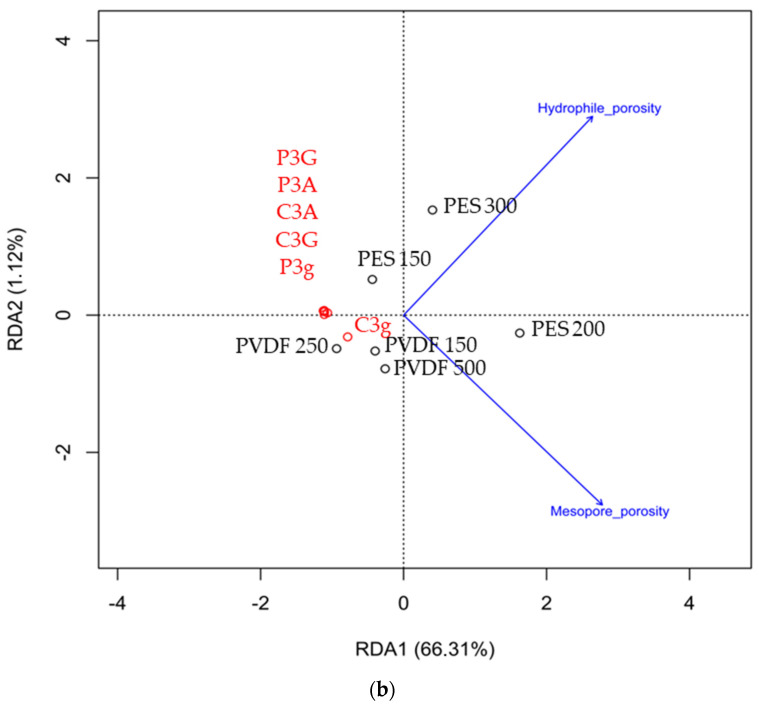
RDA biplots between total anthocyanins and organic acid migrations (**a**) or individual anthocyanins (**b**) and FM properties.

Due to the specific FM physicochemical properties of both polymers, different types of interactions may intervene between anthocyanins and FM and could explain the dissimilar migration rates. Although the compounds (anthocyanins and organic acids) are smaller than the pore sizes, interactions between them and with the membrane surface or membrane pores play a key role [35]. Thus, two RDAs were performed to find a potential correlation between membrane properties and EDFM performances in terms of total anthocyanin and organic acid migrations for the first RDA (Figure 6a) and in terms of individual anthocyanin migration for the second RDA (Figure 6b).

After permutation, collinearity diagnostic was realised as described in Section 2.4.2 and indicated that the thickness, conductivity, and contact angle variables were collinear with other explanatory variables in at least one of these datasets. These three variables were therefore removed for subsequent analyses. Then, the selection of significant FM properties on migration was carried out by stepwise approach as mentioned in Section 2.4.2. Only 2 FM properties (mesopore porosity and hydrophilic porosity) out of the nine properties were included in the total anthocyanins and total organic acid RDA, as well as in the individual anthocyanin RDA (Figure 6), while none of the properties were found for the individual organic acid RDA. For total anthocyanins and total organic acids, the RDA axes, the linear combination of these two properties, explained 49.2% of the total variation in organic acid and global anthocyanin migrations. For individual anthocyanin migration, RDA axes explained 67.4% of the variation in anthocyanin migration. RDA axes (Figure 6) explained less variation of organic acids and total anthocyanins than RDA explained the variation of individual anthocyanins (49% instead of 67%). This difference in variation explanation was mainly due to the fact that these FM properties affect the migration of the anthocyanins more than that of organic acids.

As previously mentioned [25], the cosine angle between FM properties (explanatory variable) and migration (response variables) indicated their relation. In addition, an angle below, equal, and above 90° indicates a positive, an absence and a negative correlation, respectively. For all anthocyanins in our study, both FM properties (mesopore and hydrophilic porosities) had negative correlations with them, meaning that the mesopore and hydrophilic porosities have negative impacts on anthocyanin migrations. The value of object (membrane) can also be obtained by projecting the point at a right angle on a response or explanatory variable. 

To generate predictive models based on FM properties (explanatory variables) and to define their correlation coefficient, for each anthocyanin a multivariate regression analysis was performed. A predictive model for each individual anthocyanin linked to the mesopore and hydrophilic porosities (significant FM properties) was described thanks to the general following equation:

(4) $$\ln\left(MRA\right)=\beta_{0}+\beta_{MP}.X_{MP}+\beta_{HP}.X_{HP}\;$$

where *MRA* is the migration rate of individual anthocyanins, *β*_0_ the intercept, *β_MP_* the estimated coefficient of mesopore porosity, *β_HP_* the estimated coefficient of percentage of hydrophilic pores, *X_HP_* the mean hydrophilic porosity and *X_MP_* the mean mesopore porosity for a membrane. Estimated coefficients and R^2^ for each anthocyanin’s migration are presented in Table 4. 

Interestingly, *β_MP_* the estimated coefficient of mesopore porosity was higher than the estimated coefficient of percentage of hydrophilic pores *β_HP_*_,_ indicating a stronger impact of mesopore porosity on each individual anthocyanin migration. Higher coefficient values for each property were also correlated to the anthocyanins initially most abundant in the juice, showing their higher migration rate, as demonstrated previously (Section 3.3.1).

## 4. Discussion

### 4.1. Impact of FM Properties on Anthocyanin Migration

The statistical approach allowed the determination of two main filtering layer properties impacting migration the most, which will be discussed in the following parts. However, the model explains only part of the variation in anthocyanin migration during EDFM with different FM. Thereby, to improve our understanding of anthocyanin migration performance, for both polymeric membranes, a tentative explanation will be given focusing on the impact of the membrane surface, the membrane filtrating layer and finally the backing layer. Indeed, when migrating, anthocyanins first encountered the membrane surface, which could hinder the migration, before going through the filtrating layer and then the backing layer or matrix support. Different known intermolecular interactions (electrostatic, hydrogen, hydrophobic) can occur between anthocyanins (amphipathic molecules) and the membrane polymer depending on their chemical structures, so the focus will be first placed on interactions between PES membranes and anthocyanins, then on PVDF membranes and anthocyanins [36,37,38,39]. 

#### 4.1.1. PES Membranes

**Figure 7:**
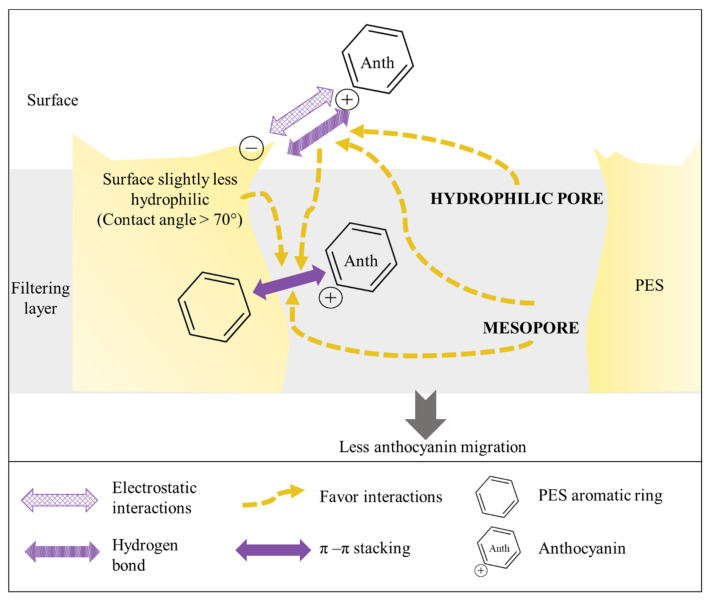
Scheme of the possible interactions which could explain PES membrane performance.

In our study, the membrane surface, at the juice’s pH, PES 150 kDa and PES 300 kDa had the highest negative zeta potential, followed by the PES 200 kDa among the membrane with a negatively charged surface (Table 3). The migration of positively charges anthocyanins has not been facilitated by membrane with moderate negatively charged surfaces, as has already been described for peptide [32]. On the contrary, the higher migration rate was obtained for the PES 150 kDa, which presented a strong negative surface charge. Even if electrostatic interactions did not help the migration, results for the desorption with a 0.1 M NaOH solution (detailed in the Supplementary Material Section, Figure S4) showed that PES 150 kDa and PES 300 kDa (with the highest negative zeta potential) had the best desorption of polyphenols, confirming electrostatic interactions between anthocyanins and the membrane. A facilitated migration by electrostatic interactions could be minimized by the impact of other stronger interactions, which hampered the migration. Concerning roughness (Rz) value, a previous study on peptides has demonstrated that small molecules (450–500 Da), like anthocyanins, could be entrapped in valley region of rough surfaces and more drag force will be necessary to detach them from FM due to electrostatic attraction between opposite charges [25]. In our study, PES 150 kDa and PES 200 kDa membranes exhibited similar Rz, which should hinder migration by trapping anthocyanins (Table 3). Surprisingly, PES 150 kDa performed well in terms of anthocyanin migration, but higher contact angle (and consequently less hydrophilic membrane) seems directly correlated to a decline in anthocyanin migration rate. Indeed, PES 200 kDa exhibited the highest contact angle among the three PES membranes and the lowest migration rate (Table 3). Thus, for PES membranes, a contact angle below 70° seems to favor anthocyanin migration (Figure 7). A membrane surface slightly less hydrophilic could tend to favor π–π (stacking) interactions between anthocyanins themselves and with PES aromatic rings (Figure 7). Thus, results showed that PES membrane surface impacted the preferential migration of anthocyanins but interactions inside the filtrating layer would have the strongest impact, as confirmed by the RDA results. 

The RDA highlighted two filtrating layer membrane properties (mesopore porosity and hydrophilic porosity) out of the nine to be correlated to individual anthocyanin migration. Indeed, one of the highest migration rates reported was obtained when PES 150 kDa was used, unlike PES 200 kDa and PES 300 kDa, and among PES membranes PES 150 kDa had the smallest percentage of hydrophilic pores and was the most performant in terms of anthocyanin’s migration (Table 3). Thus, a high percentage of hydrophilic pores in the filtrating layer tends to favor interaction between hydrophilic units of the PES polymer and the hydroxyl groups of anthocyanins, which then favor the π–π (stacking) interactions between anthocyanins itself and with PES aromatic rings [36,38,40] (Figure 7). Together, all these interactions will not favor the anthocyanin’s migration (Figure 7). Concerning mesopore porosity, for PES membranes, anthocyanin migration has been hindered by mesopores. PES 150 kDa presented mesopore porosity three times lower than PES 200 kDa and was one of the best performant membranes in terms of anthocyanin’s migration. Indeed, PES 200 kDa (with the worse performance in terms of anthocyanin migration) exhibited the highest percent of mesopores (17.3 ± 5.2 cm^3^/cm^3^) and this percentage was significantly different compared to PES 150 kDa and 300 kDa. In addition, the PES 200 kDa presented micropores. Results for desorption with a 0.1 M NaOH solution (detailed in Supplementary Material Section, Figure S5) confirmed the percentage of mesopore and the percentage of hydrophilic porosity explained the different quantity of polyphenol retrieved. Thus, the results suggested that micropores and hydrophilic porosity and in a more correlated way mesopores (Figure 6b) could hinder anthocyanin migration by increasing the contact surface between the anthocyanins and the membrane material, favouring anthocyanin and membrane interactions (Figure 7).

Finally, for PES membrane, the backing layer could also be involved in the membrane performance in terms of anthocyanin migration, but probably in a lesser extent than filtrating layer. Indeed, unlike PES 200 kDa and PES 300 kDa, PES 150 kDa was from another supplier and the backing layer was made of polypropylene instead of polyester. Since polypropylene is more hydrophobic than polyester, PES 150 kDa exhibiting the highest migration rate could have been helped by the backing layer (decreasing potential anthocyanin and membrane interactions due to its higher hydrophobicity), compared to PES 300 kDa exhibiting similar properties (but with one of the worst performances in terms of anthocyanin migration). 

#### 4.1.2. PVDF Membranes

**Figure 8:**
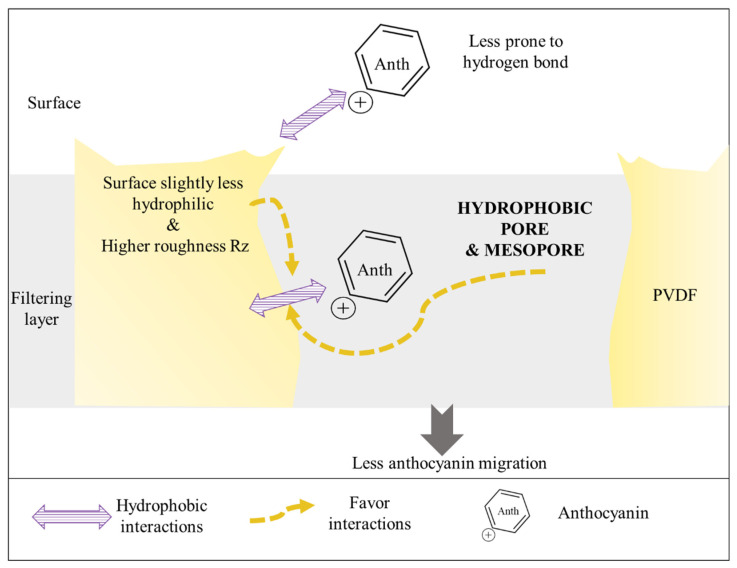
Scheme of the possible interactions which could explain PVDF membrane performance.

For PVDF membranes, contrary to PES membranes, in terms of membrane surface properties, PVDF 150 kDa and PVDF 250 kDa presented slightly positive and neutral surface charges, respectively. PVDF 500 displayed an intermediate negative charge (Table 3). The highest migration rate was obtained for the PVDF 250 kDa, which presented a surface charge close to 0 mV. It has been noticed that membrane with a positively charged surface leads to repulsive interactions between membranes and positively charged anthocyanins and thus hinders their migration [32]. However, the actual narrow range of zeta potential for PVDF membrane in the present study did not allow full explanation of the migration. Indeed, results for the desorption with 0.1 M NaOH solution (detailed in the Supplementary Material Section, Figure S4) showed that PVDF 250 kDa and PVDF 150 kDa had positive or neutral potential zeta, and the worse desorption of polyphenols, indicating that electrostatic interactions were less present. For roughness (Rz) data, the PVDF results are in accordance with the hypothesis that small molecules like anthocyanins (450–500 Da) could be entrapped in valley region of rough surfaces [25]. Indeed, PVDF 250 kDa exhibited the best performance and one of the lowest Rz (Figure 8). Regarding the contact angle, the highest migration rate was obtained when PVDF 250 kDa was used and contact angle was one of the lowest (64°) out of the six membranes. The higher contact angle was for PVDF 150 kDa (78°) and could explain the highest migration rate of PVDF 250 kDa. However, PVDF 250 kDa and PVDF 500 kDa exhibited similar contact angle but PVDF 500 kDa has the lowest migration rate among the PVDF membrane (Table 3). Thus, surface properties alone are not enough to explain the migration differences and filtrating layer properties are of main importance.

According to RDA results, hydrophilic and mesopore porosity, two main properties of the filtrating layer, explained 67.4% of the anthocyanin migration variation. It is important to highlight that all hydrophilic porosities of PVDF membranes are below the PES ones (Table 3). PVDF (a symmetrical arrangement of hydrogen and fluorine atoms around the polymeric chain [41]) are less prone to hydrogen bonding, Van der Waals interactions and π-π stacking). Consequently, PVDF membranes have a better performance than PES membranes, except for PES 150 kDa. PVDF 250 kDa, which allowed the highest migration rate, displayed around 50% of hydrophilic pores. PVDF 500 kDa displayed the lowest hydrophilic porosity compared to the PVDF 250 kDa and PVDF 150 kDa and was the less performant among the PVDF membranes. A low hydrophilic porosity seems to hinder the anthocyanin migration by increasing the chance of hydrophobic interaction (Figure 8). In the same way as for PES membrane, for PVDF membranes anthocyanin migrations have been hindered by mesopores. Results for the desorption with a 0.1 M NaOH solution (detailed in Supplementary Material Section, Figure S5) confirmed that membrane parameter (hydrophilic porosity) and other interactions (hydrophobic) caused fouling to the main extent, rather than electrostatic interactions, especially for PVDF membrane. Thus, the results suggested that hydrophilic porosity and, in a more correlated way, mesopores (Figure 6b) could hinder anthocyanin migration by increasing the contact surface between the anthocyanins and the membrane material, favouring potential anthocyanin and membrane interactions (Figure 8).

Since all PVDF membranes are made of polyester as backing layer, its properties would have influenced anthocyanin migration in the same way.

### 4.2. Impact of FM Properties on Organic Acid Migration

Only two FM properties were included in the total anthocyanins and total organic acid RDAs and were the same two properties for individual anthocyanins (Figure 6). None of the properties were included in the individual organic RDA. These results suggested that the two FM properties were correlated mostly to anthocyanin migration and not organic acid migration. Even so, due to the FM physicochemical properties, electrostatics interactions materialized between organic acids and FM could explain the dissimilar migration rate between PVDF 150 kDa and PES 200 kDa. The zeta potential of PVDF 150 kDa was positive, whereas that for PES 200 kDa was negative. PVDF 150 kDa was the only membrane exhibiting a positive zeta potential and was the one with the highest organic acid migration. It seemed that a positive zeta potential would favor the organic acid migration. At the juice’s pH, organic acids were in their anionic forms. So, positive charges’ surface membranes would help the migration of organic acid. The average roughness Rz was higher for the PES 200 kDa than PVDF 150 kDa. Since organic acids migrated from the 300 mL compartment to the 9 L compartment and the filtration layer of the anthocyanins was placed towards the anode, the migration of organic acid faced the anode. So, organic acids first crossed the support layer before the filtrating layer. Thus, the average roughness of the surface of the filtrating layer should not affect the crossing of the membrane. The hydrophilic porosity was significatively higher for PES 200 kDa compared to PVDF 150 kDa. The results suggested that a high volume of hydrophilic pores could hinder organic acid migration through membranes. PVDF 150 kDa has a volume of mesopore inferior to the PES 200 kDa which diminished the surface interaction and helped the migration.

## 5. Conclusions

To the best of our knowledge, this is the first study that demonstrated the correlation between the physicochemical properties of filtration membranes and the migration of anthocyanins and organic acids during EDFM. The full characterization of the membranes tested in EDFM system allowed classification of the performance of membranes in terms of anthocyanin migration: the best performance was obtained when PVDF 250 kDa membrane (conductivity =12.8 mS/cm, thickness = 0.218 mm, zeta potential = −0.5 mV, contact angle = 64°, roughness Rz = 8.6 μm, hydrophilic porosity = 51%, mesopores = 9.3%) was used in the EDFM system. Moreover, organic acid migration toward the anode occurred with PVDF 150 kDa (conductivity = 9.0 mS/cm, thickness = 0.228 mm, zeta potential = 3.4 mV, angle de contact = 78°, roughness Rz = 11.3 μm, hydrophilic porosity = 60%, mesopores = 7.5%) making it the most performant in that respect. pH, color, Brix° of raw and enriched juices were not altered by the 180 min EDFM treatment. These results confirmed the potential of this technology and brought a better understanding on anthocyanin and polymer interactions.

It appears from the results that the membrane properties having a significant effect on anthocyanin and total organic acid migrations were the porosity of mesopore and the hydrophilic porosity. None of these membrane properties were selected for the individual migration of organic acids, suggesting that these two FM properties are mostly correlated to anthocyanins but not organic acid migration. Correlations acquired from RDA and regression analysis suggested that the selective migration of individual anthocyanins was primarily due to intermolecular interaction between anthocyanins and membranes surfaces/pores but also membrane’s pore size. Furthermore, the obtained statistical models indicated that the migration of individual anthocyanins through membranes decreased with the percentage of hydrophilic pores and, in a more correlated way, with an increase in the porosity of mesopore. Finally, our results suggested that it is a combination of intermolecular interaction (hydrogen bond, hydrophobic interactions, π-π stacking) that may lead to strong binding in a cooperative or synergistic mode. 

However, these results are limited to the type of membranes tested. Indeed, the type of polymer impacts the membrane properties and anthocyanin migration but those selected were only commercial membranes and available at large scale for industrial uses. From a scientific point of view, whatever are available at large scale for industrial purpose, more membranes with different physicochemical characteristics could be tested to further validate the outcome of the model. The EDFM process could also be improved to enrich the juice in other polyphenols known to have a positive impact on the microbiota. Finally, to understand even better what could hinder anthocyanin migration, we are currently studying anthocyanins’ fouling mechanisms according to the membrane physicochemical properties.

## Figures and Tables

**Table 1:** Physicochemical properties of cranberry juice (* Results are reported as, L (Luminescence or lightness), a (intensity of color varying from red to green) and b (intensity of color varying from yellow to blue)).

**Colorimetry**	
L*	28.03 ± 1.13
a*	0.59 ± 0.55
b*	0.90 ± 0.39
**Total soluble solids (°Brix)**	8.2 ± 0.1
**pH**	2.53 ± 0.02
**Conductivity (mS/cm)**	2193 ± 47
**Anthocyanins (mg/L)**	
Peonidin-3-galactoside	68.56 ± 0.30
Cyanidin-3-galactoside	46.32 ± 0.17
Peonidin-3-arabinoside	28.15 ± 0.3
Cyanidin-3-arabinoside	45.01 ± 0.28
Peonidin-3-glucoside	7.87 ± 0.06
Cyanidin-3-glucoside	1.54 ± 0.06
**Proanthocyanidins (mg/L)**	
Monomers	38.70 ± 1.55
2–3 m	106.81 ± 0.885
4–5 m	17.16 ± 0.15
6–7 m	7.10 ± 0.35
>7 m	0.00 ± 0.00
**Organic Acid (mg/L)**	
Malic Acid	7812.81 ± 85.76
Citric Acid	15,294.36 ± 204.34
Quinic Acid	12,412.19 ± 58.73

**Table 2:** FM tested in EDFM configuration.

Constitutive Material	Backing Material	Cut-Off (kDa)	Suppliers	Origin
PES *	Polypropylene	150	Microdyn-Nadir (Sterlitech)	Auburn, WA, USA
PES	Non-Woven Polyester	200	Synder	Vacaville, CA, USA
PES	Non-Woven Polyester	300	Synder	Vacaville, CA, USA
PVDF *	Non-Woven Polyester	150	Microdyn-Nadir (Sterlitech)	Auburn, WA, USA
PVDF	Non-Woven Polyester	250	Synder	Vacaville, CA, USA
PVDF	Non-Woven Polyester	500	Synder	Vacaville, CA, USA

* PES = Polyether-sulfone and PVDF = Polyvinylidene fluoride.

**Table 3:** Physicochemical properties of filtration membranes measured prior EDFM.

		Membrane Properties											
		Conductivity * (mS/cm)	Thickness ** (mm)	Zêta Potential (mV)	Contact Angle (◦)	Roughness (Ra, μm)	Roughness (Rz, μm)	% Hydrophilic Porosity	Volumetric Porosity (cm^3^/cm^3^)	Porosity of Mesopore (cm^3^/cm^3^)	% of Micropores in the Membranes	% of Mesopores in the Membranes	% of Macropores in the Membranes
**Membranes**	PVDF 500 kDa	12.2 ± 1.7 ^ab^	0.224 ± 0.005 ^a^	−3.7 ± 0.7 ^bc^	65 ± 3 ^abc^	1.19 ± 0.10 ^a^	11.9 ± 2.0 ^b^	39 ± 7 ^c^	0.64 ± 0.00 ^a^	0.077 ± 0.012 ^ab^	0 ^b^	11.9 ± 1.9 ^ab^	88 ± 2 ^a^
	PVDF 250 kDa	12.8 ± 0.2 ^a^	0.218 ± 0.005 ^a^	−0.5 ± 1.0 ^ab^	64 ± 7 ^bc^	0.83 ± 0.31 ^a^	8.6 ± 2.0 ^b^	51 ± 7 ^bc^	0.63 ± 0.02 ^a^	0.058 ± 0.011 ^bc^	0.331 ± 0.573 ^b^	9.3 ± 2.0 ^b^	90 ± 2 ^a^
	PVDF 150 kDa	9.0 ± 0.3 ^ab^	0.228 ± 0.002 ^a^	3.4 ± 1.2 ^a^	78 ± 6 ^a^	1.27 ± 0.30 ^a^	11.3 ± 1.8 ^b^	60 ± 13 ^b^	0.65 ± 0.06 ^a^	0.049 ± 0.008 ^bc^	0 ^b^	7.5 ± 1.3 ^b^	93 ± 1 ^a^
	PES 300 kDa	11.6 ± 0.6 ^ab^	0.193 ± 0.005 ^b^	−11.1 ± 3.1 ^d^	57 ± 5 ^c^	1.13 ± 0.41 ^a^	11.3 ± 2.8 ^b^	99 ± 6 ^a^	0.65 ± 0.07 ^a^	0.048 ± 0.016 ^bc^	0 ^b^	7.5 ± 2.2 ^b^	93 ± 2 ^a^
	PES 200 kDa	11.5 ± 0.4 ^ab^	0.181 ± 0.005 ^b^	−5.2 ± 1.5 ^c^	72 ± 1 ^ab^	1.46 ± 0.53 ^a^	18.6 ± 3.7 ^a^	92 ± 1 ^a^	0.64 ± 0.03 ^a^	0.110 ± 0.030 ^a^	3.718 ± 0.829 ^a^	17.3 ± 5.2 ^a^	79 ± 6 ^b^
	PES 150 kDa	7.6 ± 0.3 ^b^	0.224 ± 0.004 ^a^	−10.3 ± 0.7 ^d^	61 ± 4 ^bc^	1.38 ± 0.36 ^a^	17.0 ± 2.2 ^a^	87 ± 4 ^a^	0.60 ± 0.02 ^a^	0.032 ± 0.000 ^c^	1.220 ± 0.625 ^b^	5.4 ± 0.5 ^b^	93 ± 1 ^a^
***p*-value**		*p* = 0.018	*p* < 0.001	*p* < 0.001	*p* = 0.002	*p* = 0.127	*p* < 0.001	*p* < 0.0001	*p* = 0.887	*p* = 0.001	*p* < 0.0001	*p* = 0.0015	*p* = 0.003

Values followed with different letters (a, b, c, d) for the same column are statistically significantly different (Tukey test) at *p* < 0.05. * Test with rank transformation ** The statistical test was applied to transformed data

**Table 4:** Significant estimated coefficients for individual anthocyanin’s migration.

	$\beta_{0}$	$\beta_{MP}$	$\beta_{HP}$	R^2^
Peonidin-3-galactoside	2.084389	−9.440033	−0.010597	0.4622338
Cyanidin-3-galactoside	1.381813	−6.378894	−0.006691	0.4263189
Peonidin-3-arabinoside	1.035268	−4.949934	−0.005111	0.4697571
Cyanidin-3-arabinoside	1.391271	−6.573328	−0.006869	0.4615215
Peonidin-3-glucoside	0.231830	−0.967541	−0.001180	0.4795030
Cyanidin-3-glucoside	0.045675	−0.138578	−0.000302	0.4561404

## Data Availability

The original contributions presented in the study are included in the article and Supplementary Material, further inquiries can be directed to the corresponding author.

## Supplementary Materials

The following supporting information can be downloaded at: https://www.mdpi.com/article/10.3390/membranes14050111/s1, Figure S1: Conductivity evolution of raw (a) and enriched juice (b) as a function of time during EDFM for each tested membrane. Figure S2: pH evolution of enriched (a) and raw juice (b) as a function of time during EDFM for each tested membrane. Figure S3: Evolution of total PACS during EDFM Treatment for each membrane tested. Figure S4: Pictures of filtrating layer of pristine membranes, after EDFM membranes and after desorption membranes with their associated L*, a*, b* values. Figure S5: Quantity (g) of polyphenols desorbed by m^2^ of membrane vs migration rates for membranes tested. Table S1: Degree brix measured for raw and treated cranberry juices before and after EDFM for each tested membrane. Table S2: Evolution of Color parameters for the final raw and enriched juices. References [7,21,24,32,42,43] are cited in Supplementary Materials.

## References

[1] Clemente-Suárez V.J., Beltrán-Velasco A.I., Redondo-Flórez L., Martín-Rodríguez A., Tornero-Aguilera J.F. (2023). Global Impacts of Western Diet and Its Effects on Metabolism and Health: A Narrative Review. Nutrients.

[2] Ruel G., Couillard C. (2007). Evidences of the Cardioprotective Potential of Fruits: The Case of Cranberries. Mol. Nutr. Food Res..

[3] Del Bo’ C., Bernardi S., Marino M., Porrini M., Tucci M., Guglielmetti S., Cherubini A., Carrieri B., Kirkup B., Kroon P. (2019). Systematic Review on Polyphenol Intake and Health Outcomes: Is There Sufficient Evidence to Define a Health-Promoting Polyphenol-Rich Dietary Pattern?. Nutrients.

[4] Fu Z., Liska D., Talan D., Chung M. (2017). Cranberry Reduces the Risk of Urinary Tract Infection Recurrence in Otherwise Healthy Women: A Systematic Review and Meta-Analysis. J. Nutr..

[5] Messaoudene M., Pidgeon R., Richard C., Ponce M., Diop K., Benlaifaoui M., Nolin-Lapalme A., Cauchois F., Malo J., Belkaid W. (2022). A Natural Polyphenol Exerts Antitumor Activity and Circumvents Anti–PD-1 Resistance through Effects on the Gut Microbiota. Cancer Discov..

[6] Caillet S., Lorenzo G., Côté J., Doyon G., Sylvain J.-F., Lacroix M. (2012). Cancer Chemopreventive Effect of Fractions from Cranberry Products. Food Res. Int..

[7] Mattioli R., Francioso A., Mosca L., Silva P. (2020). Anthocyanins: A Comprehensive Review of Their Chemical Properties and Health Effects on Cardiovascular and Neurodegenerative Diseases. Molecules.

[8] Blumberg J.B., Camesano T.A., Cassidy A., Kris-Etherton P., Howell A., Manach C., Ostertag L.M., Sies H., Skulas-Ray A., Vita J.A. (2013). Cranberries and Their Bioactive Constituents in Human Health. Adv. Nutr..

[9] Liu J., Hao W., He Z., Kwek E., Zhu H., Ma N., Ma K.Y., Chen Z.-Y. (2021). Blueberry and Cranberry Anthocyanin Extracts Reduce Bodyweight and Modulate Gut Microbiota in C57BL/6 J Mice Fed with a High-Fat Diet. Eur. J. Nutr..

[10] Hair R., Sakaki J.R., Chun O.K. (2021). Anthocyanins, Microbiome and Health Benefits in Aging. Molecules.

[11] Kapoor P., Tiwari A., Sharma S., Tiwari V., Sheoran B., Ali U., Garg M. (2023). Effect of Anthocyanins on Gut Health Markers, Firmicutes-Bacteroidetes Ratio and Short-Chain Fatty Acids: A Systematic Review via Meta-Analysis. Sci. Rep..

[12] Alchera F., Ginepro M., Giacalone G. (2022). Microwave-Assisted Extraction of Polyphenols from Blackcurrant By-Products and Possible Uses of the Extracts in Active Packaging. Foods.

[13] Louli V., Ragoussis N., Magoulas K. (2004). Recovery of Phenolic Antioxidants from Wine Industry By-Products. Bioresour. Technol..

[14] He L., Zhang X., Xu H., Xu C., Yuan F., Knez Ž., Novak Z., Gao Y. (2012). Subcritical Water Extraction of Phenolic Compounds from Pomegranate (*Punica granatum* L.) Seed Residues and Investigation into Their Antioxidant Activities with HPLC–ABTS+ Assay. Food Bioprod. Process..

[15] Ma Y.-Q., Chen J.-C., Liu D.-H., Ye X.-Q. (2009). Simultaneous Extraction of Phenolic Compounds of Citrus Peel Extracts: Effect of Ultrasound. Ultrason. Sonochem..

[16] Soto M.L., Moure A., Domínguez H., Parajó J.C. (2011). Recovery, Concentration and Purification of Phenolic Compounds by Adsorption: A Review. J. Food Eng..

[17] Li B.B., Smith B., Hossain M.M. (2006). Extraction of Phenolics from Citrus Peels. Sep. Purif. Technol..

[18] Ignat I., Volf I., Popa V.I. (2011). A Critical Review of Methods for Characterisation of Polyphenolic Compounds in Fruits and Vegetables. Food Chem..

[19] Bleve M., Ciurlia L., Erroi E., Lionetto G., Longo L., Rescio L., Schettino T., Vasapollo G. (2008). An Innovative Method for the Purification of Anthocyanins from Grape Skin Extracts by Using Liquid and Sub-Critical Carbon Dioxide. Sep. Purif. Technol..

[20] Bazinet L., Doyen A. (2017). Antioxidants, Mechanisms, and Recovery by Membrane Processes. Crit. Rev. Food Sci. Nutr..

[21] Bazinet L., Cossec C., Gaudreau H., Desjardins Y. (2009). Production of a Phenolic Antioxidant Enriched Cranberry Juice by Electrodialysis with Filtration Membrane. J. Agric. Food Chem..

[22] Faucher M., Serre É., Langevin M.-È., Mikhaylin S., Lutin F., Bazinet L. (2018). Drastic Energy Consumption Reduction and Ecoefficiency Improvement of Cranberry Juice Deacidification by Electrodialysis with Bipolar Membranes at Semi-Industrial Scale: Reuse of the Recovery Solution. J. Membr. Sci..

[23] Pappas E., Schaich K.M. (2009). Phytochemicals of Cranberries and Cranberry Products: Characterization, Potential Health Effects, and Processing Stability. Crit. Rev. Food Sci. Nutr..

[24] Bazinet L., Brianceau S., Dubé P., Desjardins Y. (2012). Evolution of Cranberry Juice Physico-Chemical Parameters during Phenolic Antioxidant Enrichment by Electrodialysis with Filtration Membrane. Sep. Purif. Technol..

[25] Kadel S., Daigle G., Thibodeau J., Perreault V., Pellerin G., Lainé C., Bazinet L. (2021). How Physicochemical Properties of Filtration Membranes Impact Peptide Migration and Selectivity during Electrodialysis with Filtration Membranes: Development of Predictive Statistical Models and Understanding of Mechanisms Involved. J. Membr. Sci..

[26] Wu X., Prior R.L. (2005). Systematic Identification and Characterization of Anthocyanins by HPLC-ESI-MS/MS in Common Foods in the United States: Fruits and Berries. J. Agric. Food Chem..

[27] Khanal R.C., Howard L.R., Brownmiller C.R., Prior R.L. (2009). Influence of Extrusion Processing on Procyanidin Composition and Total Anthocyanin Contents of Blueberry Pomace. J. Food Sci..

[28] Blanchet F.G., Legendre P., Borcard D. (2008). Forward Selection of Explanatory Variables. Ecology.

[29] Legendre P., Legendre L. (2012). Complex Ecological Data Sets. Developments in Environmental Modelling.

[30] Burchett W.W., Ellis A.R., Harrar S.W., Bathke A.C. (2017). Nonparametric Inference for Multivariate Data: The R Package Npmv. J. Stat. Soft..

[31] Belsley D.A., Kuh E., Welsch R.E. (1980). Regression Diagnostics.

[32] Husson E., Araya-Farias M., Gagné A., Bazinet L. (2013). Selective Anthocyanins Enrichment of Cranberry Juice by Electrodialysis with Filtration Membrane: Influence of Membranes Characteristics. J. Membr. Sci..

[33] Cissé M., Vaillant F., Pallet D., Dornier M. (2011). Selecting Ultrafiltration and Nanofiltration Membranes to Concentrate Anthocyanins from Roselle Extract (*Hibiscus sabdariffa* L.). Food Res. Int..

[34] Serre E., Rozoy E., Pedneault K., Lacour S., Bazinet L. (2016). Deacidification of Cranberry Juice by Electrodialysis: Impact of Membrane Types and Configurations on Acid Migration and Juice Physicochemical Characteristics. Sep. Purif. Technol..

[35] Vernhet A. (2002). Fouling of Organic Microfiltration Membranes by Wine Constituents: Importance, Relative Impact of Wine Polysccharides and Polyphenols and Incidence of Membrane Properties. J. Membr. Sci..

[36] Susanto H., Feng Y., Ulbricht M. (2009). Fouling Behavior of Aqueous Solutions of Polyphenolic Compounds during Ultrafiltration. J. Food Eng..

[37] Cartalade D., Vernhet A. (2006). Polar Interactions in Flavan-3-Ol Adsorption on Solid Surfaces. J. Agric. Food Chem..

[38] Laborde B., Moine-Ledoux V., Richard T., Saucier C., Dubourdieu D., Monti J.-P. (2006). PVPP−Polyphenol Complexes: A Molecular Approach. J. Agric. Food Chem..

[39] Cassano A., De Luca G., Conidi C., Drioli E. (2017). Effect of Polyphenols-Membrane Interactions on the Performance of Membrane-Based Processes. A Review. Coord. Chem. Rev..

[40] Huang J., Huang K., Liu S., Luo Q., Xu M. (2007). Adsorption Properties of Tea Polyphenols onto Three Polymeric Adsorbents with Amide Group. J. Colloid Interface Sci..

[41] Ramakrishna S., Ma Z., Matsuura T. (2011). Polymer Membranes in Biotechnology: Preparation, Functionalization and Application.

[42] Bazinet L., Castaigne F., Pouliot Y. (2005). Relative Contribution of Proteins to Conductivity Changes in Skim Milk during Chemical Acidification. Appl. Eng. Agric..

[43] Pismenskaya N., Sarapulova V., Klevtsova A., Mikhaylin S., Bazinet L. (2020). Adsorption of Anthocyanins by Cation and Anion Exchange Resins with Aromatic and Aliphatic Polymer Matrices. Int. J. Mol. Sci..

