# Supplementary material for: Targeted Anthocyanin Enrichment of Cranberry Juice by Electrodialysis with Filtration Membranes: Impact of Filtration Membrane Physicochemical Properties and Predictive Statistical Models
Source: Membranes (Basel). 2024 May 14;14(5):111. doi: 10.3390/membranes14050111 (PMC11123422; doi:10.3390/membranes14050111)
Supplement: Supplementary file 1 [file membranes-14-00111-s001.zip › membranes-2979697-supplementary.pdf]

membranes

Supplementary Materials

Targeted Anthocyanin Enrichment of Cranberry Juice by Electrodialysis with Filtration Membranes: Impact of Filtration Membrane Physicochemical Properties and Predictive Statistical Models

Eva Revellat [1,2] and Laurent Bazinet [1,2,*]

[1] Food Sciences Department, Institute of Nutrition and Functional Foods (INAF), Université Laval, Québec, QC G1V OA6, Canada; eva.revellat.1@ulaval.ca
[2] Laboratoire de Transformation Alimentaire et Procédés ElectroMembranaires (LTAPEM, Laboratory of Food Processing and ElectroMembrane Processes), Université Laval, Québec, QC G1V OA6, Canada
* Correspondence: laurent.bazinet@fsaa.ulaval.ca

S.3.2. Physicochemical parameters of cranberry juices during EDFM

Conductivity

Whatever the juice (raw or enriched), no statistical conductivity difference ($P = 0.3033$) was observed between EDFM treatments for any FM used as shown in Figure S1. The enriched juice conductivity was slightly decreasing starting from an average conductivity of 2735 µS/cm and a final conductivity of 2595 µS/cm (Figure S1b). Such a low variation in conductivity, in the range of standard deviation, suggested that no noticeable demineralization took place with a global demineralization rate of the enriched juice reaching no more than 5.1%. Additionally, the conductivity of the raw juice increased very slightly from 2271 to 2493 µS/cm (Figure S1a). Results werecomparable with the EDFM configuration and previous studies [1] [2]. Since, K^+ and Cl^- ions electrical mobilities are supposed to be superior to anthocyanin mobilities [3], the conductivity of the enriched juice declined but the electroneutrality of the juices is maintained by the compensation of ions in solution. Indeed, in the 300 mL juice in contact with CEM, during EDMF, K^+ is the main ion migrating through this CEM while anthocyanins and K^+ ions from the 9 L raw juice migrated toward the cathode through the FM. And the Cl^- anions in the 300 mL juice migrated toward the anode through the FM to the 9 L raw juice.

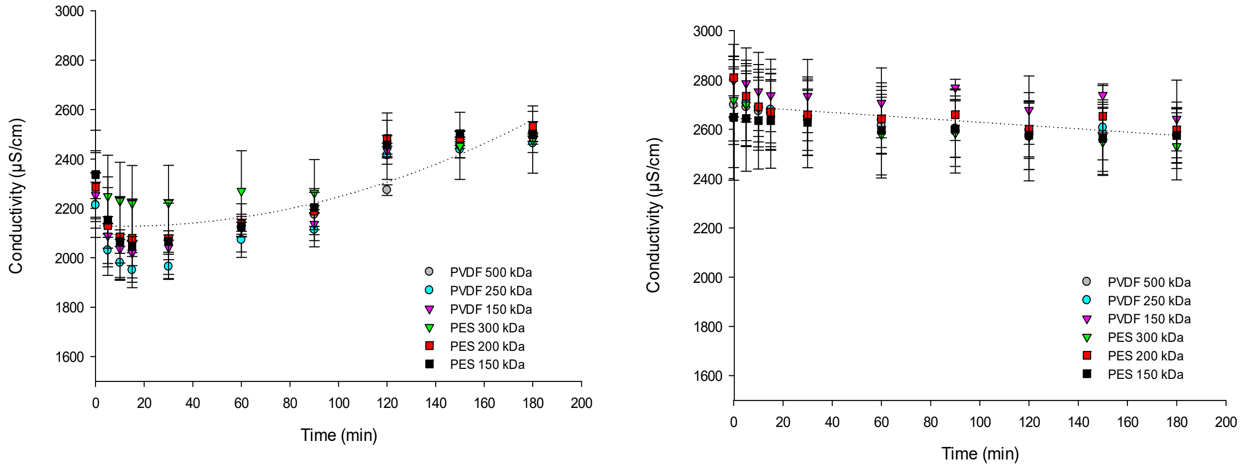

(**a**) (**b**)

Figure S1. Conductivity evolution of raw (**a**) and enriched juice (**b**) as a function of time during EDFM for each tested membrane.

pH

For the raw juice, no significant change in pH appeared during the treatment whatever the type of FM employed in the EDFM (Figure S2b). However, the final pH (2.56 ± 0.02) of the enriched juice processed through the PES 200 kDa was significatively different from the final pH of the enriched juice processed through any other membranes ($p < 0.0002$): this difference was lower than 0.05 which was in the range of the pH meter precision (Figure S2a). Thus, EDFM treatment had no significative impact on the H^+ concentration of treated juices. Also, as previously mentioned by [2], the constant pH confirmed that anthocyanins are still positively charges during the treatment, allowing their migration to the enriched juice compartment. The 9 L of raw juice were going through the system only twice which could explain the constant pH for the raw juice like previously mentioned.

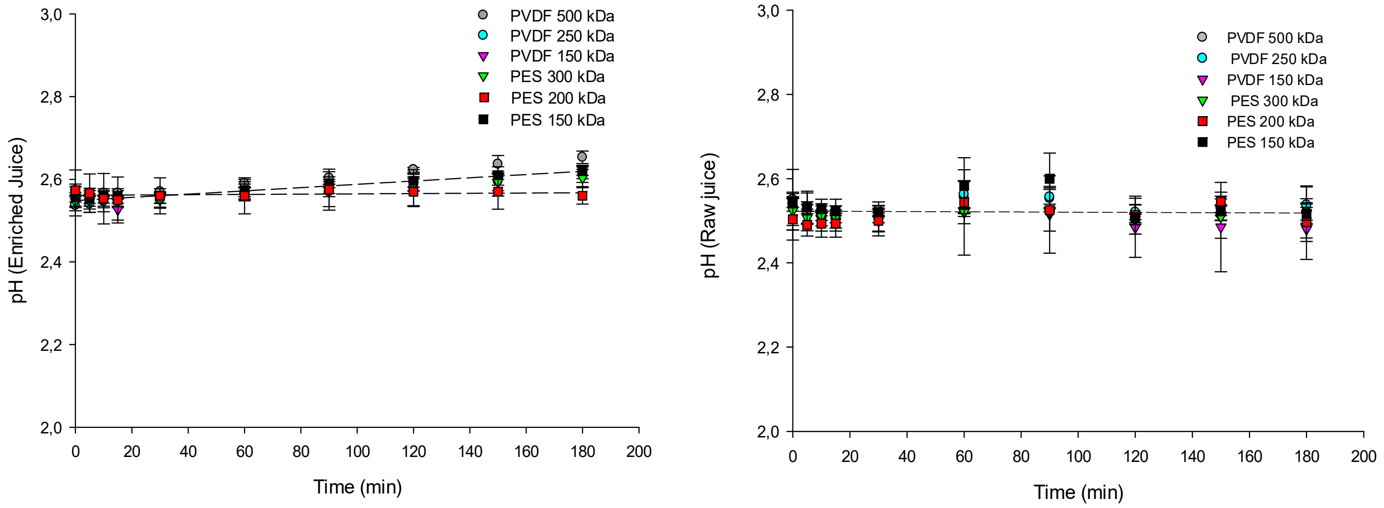

(**a**) (**b**)

Figure S2. pH evolution of enriched (**a**) and raw juice (**b**) as a function of time during EDFM for each tested membrane.

Degree Brix

Total soluble solids (sugars, minerals) in cranberry juice can be defined by the degree °Brix. No significant change in °Brix appeared between EDFM treatments for any FM (Table S1). Indeed, final degree brix of enriched juice was significatively the same for all membranes tested ($p = 0.0603$) and final degree brix of raw juice was not different for all membranes ($p = 0.1236$). Also, the t-test analysis ensured that no significant change of brix appeared after treatment. The degree °Brix value for enriched juice was lower than the treated juice because of dilution effect induced by dead volume of water present in the system as reported in the literature for small scale ED cell [1]. In addition, as noticed in a previous study, at the juice's pH (2.5-2.6), sugar molecules are not charged because the hydroxyls groups are protonated and the migration of K^+ and Cl^- compensated themselves for the mineral changes. Thus, sugar molecules did not migrate in the EDFM process (with electric field as the driving force).

Table S1. Degree brix measured for raw and treated cranberry juices before and after EDFM for each tested membrane.

Juice		PES 300 kDa	PES 200 kDa	PES 150 kDa	PVDF 500 kDa	PVDF 250 kDa	PVDF 150 kDa
Raw	Initial	8.20 ± 0.10 ^{Aa*}	8.27 ± 0.12 ^{aA**}	8.07 ± 0.15 ^{aA}	7.97 ± 0.32 ^{aA}	8.33 ± 0.23 ^{aA}	8.33 ± 0.15 ^{aA}
	Final	8.20 ± 0.10 ^{aA}	8.20 ± 0.10 ^{aA}	8.17 ± 0.12 ^{aA}	7.90 ± 0.20 ^{aA}	8.10 ± 0.10 ^{aA}	8.20 ± 0.10 ^{aA}
Enriched	Initial	6.97 ± 0.49 ^{aA}	7.23 ± 0.06 ^{aA}	7.27 ± 0.31 ^{aA}	7.20 ± 0.46 ^{aA}	7.07 ± 0.15 ^{aA}	7.23 ± 0.06 ^{aA}
	Final	6.93^{aA} ± 0.38	7.07 ± 0.15 ^{aA}	6.87 ± 0.21 ^{aA}	6.60 ± 0.20 ^{aA}	6.93 ± 0.38 ^{aA}	7.13 ± 0.25 ^{aA}

* For a same juice, values followed with same letter (a) for each column are not statistically significantly different (Tukey test) at p < 0.05. ** For a same juice, values followed with same letter (A) for the same line are not statistically significantly different (Tukey test) at p < 0.05.

Color

For both treated juices, the color parameters (L*a*b) were not significantly different regardless of the type of FM nor significantly different from the control juice parameters (Table 1 and Table S2). At low pH (<3), anthocyanins were present as a red to orange flavylium cations [4]. Thus, none of color parameters have been affected by the process.

Table S2. Evolution of Color parameters for the final raw and enriched juices.

	PES 300 kDa			PES 200 kDa			PES 150 kDa			PVDF 500 kDa			PVDF 250 kDa			PVDF 150 kDa		
**Juice**	*L*	*a*	*b*	*L*	*a*	*b*	*L*	*a*	*b*	*L*	*a*	*b*	*L*	*a*	*b*	*L*	*a*	*b*
**Final enriched**	28.26 ± 0.17 ^{Aa*}	0.42 ± 0.47 ^{Aa*}	0.97 ± 0.33 ^{Aa*}	28.69 ± 0.01 ^{Aa*}	0.97 ± 0.11 ^{Aa*}	0.67 ± 0.06 ^{Aa*}	28.42 ± 0.09 ^{Aa*}	0.38 ± 0.26 ^{Aa*}	0.72 ± 0.03 ^{Aa*}	28.51 ± 0.12 ^{Aa*}	0.61 ± 0.21 ^{Aa*}	0.80 ± 0.08 ^{Aa*}	28.39 ± 0.00 ^{Aa*}	0.91 ± 0.54 ^{Aa*}	0.58 ± 0.27 ^{Aa*}	28.44 ± 0.22 ^{Aa*}	0.70 ± 0.15 ^{Aa*}	0.72 ± 0.06 ^{Aa*}
**Final raw**	28.35 ± 0.16 ^{Aa*}	0.22 ± 0.45 ^{Aa*}	0.90 ± 0.27 ^{Aa*}	28.38 ± 0.12 ^{Aa*}	0.90 ± 0.11 ^{Aa*}	0.73 ± 0.15 ^{Aa*}	28.53 ± 0.24 ^{Aa*}	0.47 ± 0.30 ^{Aa*}	0.82 ± 0.07 ^{Aa*}	28.27 ± 0.03 ^{Aa*}	0.53 ± 0.38 ^{Aa*}	0.73 ± 0.08 ^{Aa*}	28.12 ± 0.19 ^{Aa*}	1.09 ± 0.65 ^{Aa*}	0.55 ± 0.26 ^{Aa*}	28.13 ± 0.34 ^{Aa*}	0.84 ± 0.12 ^{Aa*}	0.77 ± 0.08 ^{Aa*}

* For a same juice, values (L*, a* or b*) followed with same letter (a) for each column are not statistically significantly different (Tukey test) at p < 0.05. ** For a same juice, values (L*, a* or b*) followed with same letter (A) for the same line are not statistically significantly different (Tukey test) at p < 0.05.

S.3.3.2. PAC migration

PAC concentrations in both compartments for all the FMs were determined by HPLC and global PAC concentrations are presented in Figure S3. Data suggested that membrane materials and MWCOs in the EDFM conditions tested did not allow the migration of PACs to the 300 mL compartment whatever the membranes tested (P = 0.9025). The same applied for the raw juice (P = 0.4722) with an initial concentration of 187.8 ± 4.8 mg/L and a final concentration of 188.5 ±2.6 mg/L (Data not shown). Bazinet et al. [5] have shown a possible migration of the PACs toward a 300 L compartment after 4 hours of treatment, and this migration was not visible if the treatment was shorter (2h). However, in the present study, even with a longer treatment (3h), no significative variation in PACs concentration was noticed. This can be explained by the facts that anionic and cationic membranes as well as the FM used in past studies were not similar to those employed here. Indeed, cationic/anionic membranes wereless conductive in our study as shown by the higher average initial global resistance of the system compared to the previous study (78.2 ± 5.2 Ω instead of 69.5 ± 5.0 Ω respectively). The configuration was also different; indeed, the raw juice was circulated 60 times (450 mL) instead of twice here, leading to anthocyanin's impoverishment of the raw juice. These results suggest that an anthocyanins impoverishment of the raw juice would be necessary to allow PAC migration. This also supported the previous observation concerning the anthocyanin concentration-based migration (section 3.3.1). The fast depletion of anthocyanins in cranberry juice may accelerate PAC migration, since less molecules are available for migration.

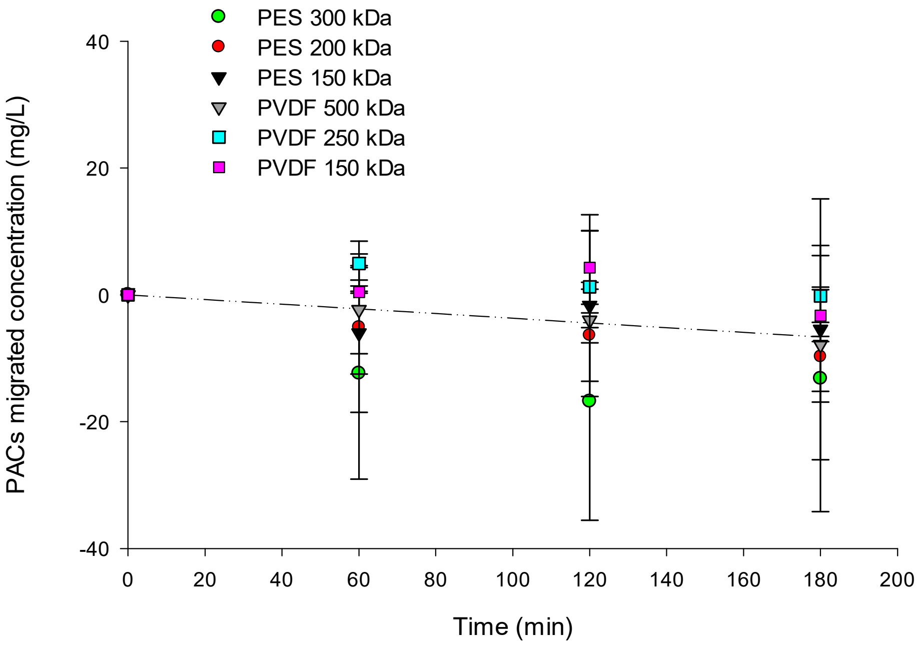

Figure S3. Evolution of total PACS during EDFM Treatment for each membrane tested.

S.4.1.1. PES membranes and S.4.1.2. PVDF Membranes

Surprisingly, electrostatic interactions which could have facilitated migrations were not highlighted: their positive impact on migration could have been minimized by other interactions hampering the migration. Hence, complementary tests were done to assess or not electrostatic interactions. After EDFM, membranes were soaked in 10 mL of 0.1M NaOH solution for 10 minutes. Thus, electric charge of anthocyanin changed because of the pH shift induced by the NaOH solution [6]. Anthocyanins were positively charged at pH of the juice but became negatively charged at basic pH. Anthocyanins adsorbed on membranes by electrostatic interactions could have been detached.

Filtrating layer color and desorption

After EDFM treatment, a light red coloration was observed on PES membranes suggesting that only a moderate amount of anthocyanins was trapped or adsorbed on the surface of PES membranes (Figure S4) whereas a strong red coloration was observed on PVDF membranes. In contact with the 0.1M NaOH solution, membranes and then the desorption solutions acquired a blue-green coloration characteristic of anthocyanin molecular state at basic pH [4].

The color of the membranes 1) after EDFM and 2) after desorption was indicated via the L*a*b* system, a three-dimensional color representation system (Figure S4). L*, a* and b* values were different for all membranes after EDFM and after desorption (Tukey test). The L* values increased after desorption indicating that membrane waswhiter. After desorption, the a* value decreased for each membrane and the b* value increased for each membrane but not for the PES 200 and PES 300. a* parameter is on the red-green axis pairs of opposite colors, (+a* in the direction of red; -a* in the direction of green) so a a* value decreasing for all membranes meant that membranes were less red. The b* parameter is placed on the yellow-blue axis pairs (+b* in the direction of yellow; -b* in the direction of blue), so b* value increasing for all PVDF membranes and PES 150 kDa, indicating that the membrane colors tended toward the yellow axis.

Also, to assess if the fouling has been completely removed from the membrane, a comparison of color values between the pristine membranes (control values) and the membrane after desorption was done. After membrane desorption, L* parameter values

were significantly different from value for pristine membrane (Dunnett test) for PES 200 kDa ($p=0.003$), PVDF 250 kDa ($p<0.001$), and PVDF 150 kDa ($p<0.001$). Also, after desorption, a* parameter values were significantly different from control value (Dunnett test) for all membranes PVDF membranes but not for PES membranes. Finally, b* parameter values were significantly different from control values (Dunnett test) for all membranes except PES 300 kDa ($p=0.135$). These results suggested that the desorption with 0.1M NaOH solution was less efficient for PVDF membranes than the PES membranes. The desorption of PVDF 250 kDa and PVDF 150 kDa were the worse one. Indeed the 3 color parameters values were significantly different from control values. The desorption of PVDF 500 kDa and PES 200 kDa were slightly better as 2 color parameters were different from control values. L* value was not different from control value for the PVDF 500 kDa and a*value was not different from control value for the PES 200 kDa. For the PES 150 kDa, the b* value was the only parameter significantly different compared to the value for the pristine membrane. The desorption of the PES 300 kDa was the most efficient as the 3 colors parameters were similar to control values.

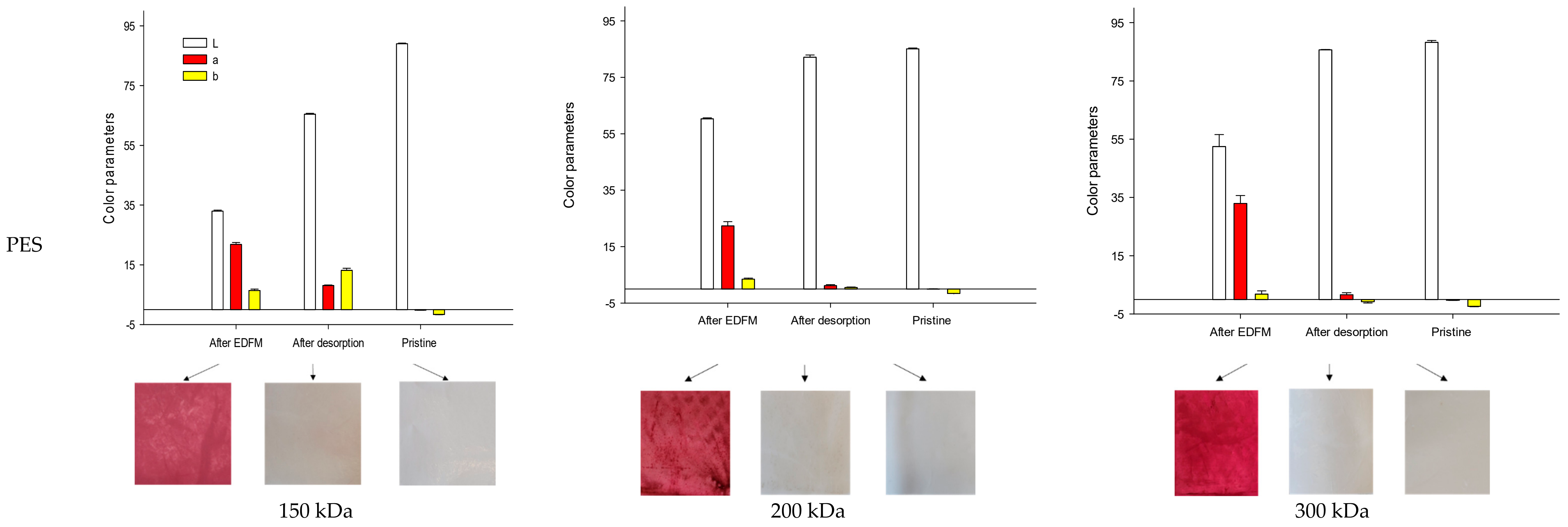
PES
Color parameters
95
75
55
35
15
-5
L
a
b
After EDFM
After desorption
Pristine
150 kDa
200 kDa
300 kDa

PVDF

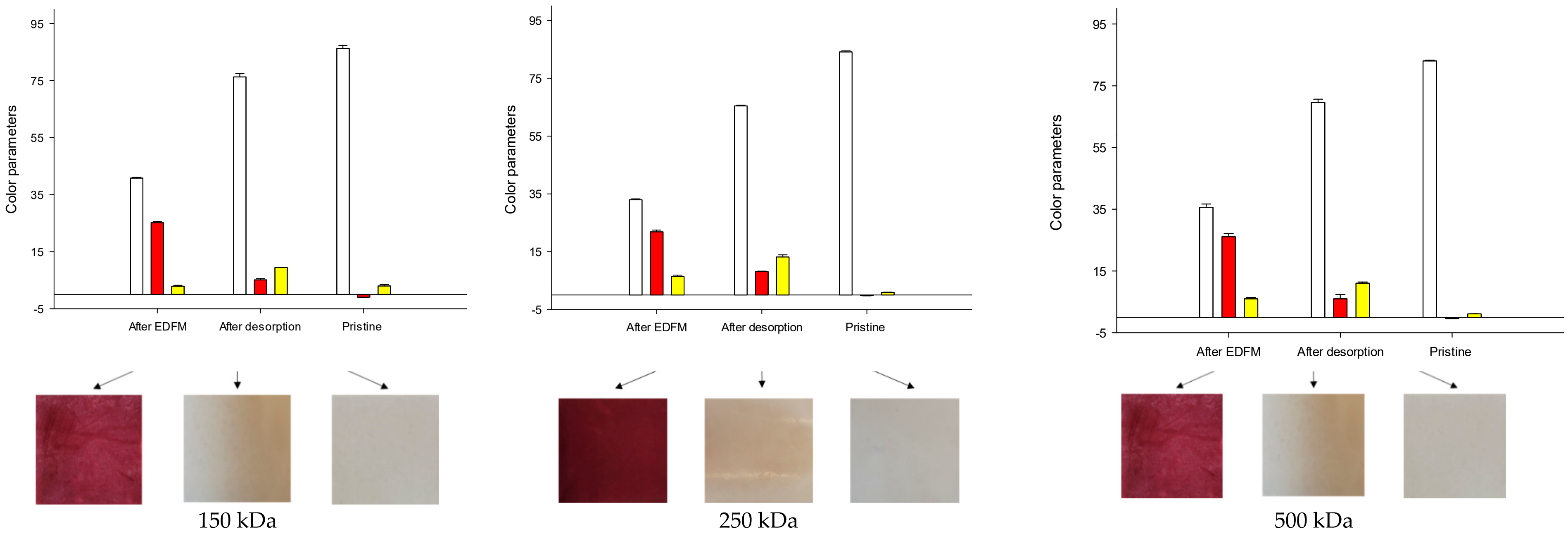

Figure S4. Pictures of filtrating layer of pristine membranes, after EDFM membranes and after desorption membranes with their associated L*, a*, b* values.

Polyphenol content in desorption solution and membrane performance

The quantities of total polyphenol retrieved from the membranes were also measured and compared to the performance of the membrane in terms of anthocyanins migration (**Error! Reference source not found.**S5).

For PES membranes, the quantity of polyphenol retrieved in the solution was the lowest (especially for the PES 150 kDa), but the desorption of the membrane was different according to the membrane. PES 300 kDa got back to pristine color values after desorption whereas for the PES 150 kDa, two color parameters after desorption were similar to pristine membranes and only one color parameter was similar to the pristine membrane for the PES 200 kDa. These results could suggest that :1) For the PES 200 kDa, more polyphenols were entrapped in membrane's pores, which was in accordance with the high mesopore porosity of the PES 200 kDa. Indeed, a membrane with more mesopores led to more interaction in the pores, explaining that all polyphenols had not been desorbed from it after desorption. Interestingly, the amount of desorbed polyphenols was similar for the PES 300 kDa but the low mesopore porosity of the PES 300 kDa facilitated the desorption of polyphenols from the membrane. Indeed, a membrane with more macropores led to less interactions in the pores. Also, as PES 300 kDa regained pristine color after desorption, these results suggested that most of the interactions between the membrane and the polyphenols were electrostatic interactions. 2) For PES 150 kDa, the low amount of polyphenol retrieved suggested first that the amount of polyphenol entrapped in the PES 150 kDa was low but as the color of the membrane was different from the pristine one, the results suggested that other type of interaction were involved between anthocyanins and the membrane (hydrophobic interaction) which was in accordance with the lowest hydrophilic porosity of the membrane.

For PVDF membranes, polyphenol concentrations retrieved in the solutions were the highest, but desorption of the membrane was one of the less efficient (color parameters after desorption were different to the control membranes). These results could suggest :1) A remaining part of anthocyanins was still entrapped in membranes, and the pH change did not allow to remove them all from the pores because other types of interactions could be involved in the fouling, 2) A high amount of polyphenols was adsorbed or entrapped in the membrane and have been retrieved from it but not all of them have been retrieved after 10 mins of soaking in the desorption solutions. Electrostatic interactions seemed to be involved in the desorption of PVDF membranes but to a lesser extent than other interactions (hydrophobic interactions). Indeed, the most performant membrane in terms of anthocyanin migration (PVDF 250 kDa) was also the less cleaned by the desorption solution but also the one with the highest amount of total polyphenol retrieved from it. Zeta potential values of PVDF were from -3.7 mV (PVDF 500 kDa) to 3.4 mV (PVDF 150 kDa). PVDF 150 kDa and PVDF 500 kDa have a similar polyphenol amount retrieved from the membrane but PVDF 150 kDa with a positive zeta potential was also less cleaned than PVDF 500 kDa. These results suggested again that membrane parameter (hydrophilic porosity) and other interactions (hydrophobic) caused fouling in a main extent than electrostatic interactions.

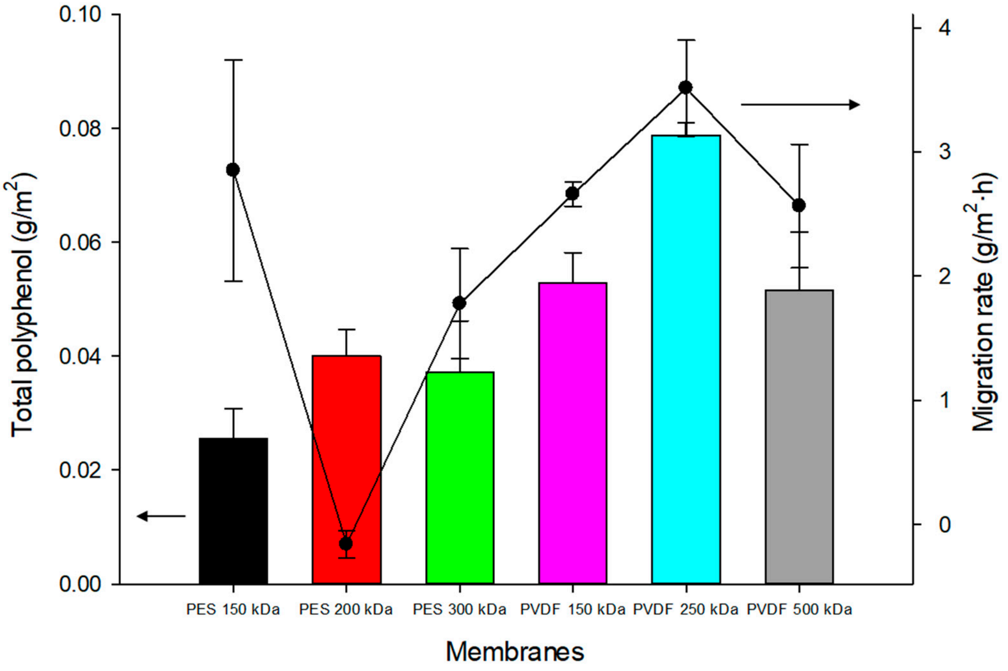

Figure S5. Quantity (g) of polyphenols desorbed by m² of membrane vs migration rates for membranes tested.

References

1. Bazinet, L.; Brianceau, S.; Dubé, P.; Desjardins, Y. Evolution of Cranberry Juice Physico-Chemical Parameters during Phenolic Antioxidant Enrichment by Electrodialysis with Filtration Membrane. *Sep. Purif. Technol.* **2012**, *87*, 31–39, doi:10.1016/j.seppur.2011.11.017.
2. Husson, E.; Araya-Farias, M.; Gagné, A.; Bazinet, L. Selective Anthocyanins Enrichment of Cranberry Juice by Electrodialysis with Filtration Membrane: Influence of Membranes Characteristics. *J. Membr. Sci.* **2013**, *448*, 114–124, doi:10.1016/j.memsci.2013.06.061.
3. L. Bazinet; F. Castaigne; Y. Pouliot Relative Contribution of Proteins to Conductivity Changes in Skim Milk during Chemical Acidification. *Appl. Eng. Agric.* **2005**, *21*, 455–464, doi:10.13031/2013.18441.
4. Mattioli, R.; Francioso, A.; Mosca, L.; Silva, P. Anthocyanins: A Comprehensive Review of Their Chemical Properties and Health Effects on Cardiovascular and Neurodegenerative Diseases. *Molecules* **2020**, *25*, 3809, doi:10.3390/molecules25173809.
5. Bazinet, L.; Cossec, C.; Gaudreau, H.; Desjardins, Y. Production of a Phenolic Antioxidant Enriched Cranberry Juice by Electrodialysis with Filtration Membrane. *J. Agric. Food Chem.* **2009**, *57*, 10245–10251, doi:10.1021/jf9021114.
6. Pismenskaya, N.; Sarapulova, V.; Klevtsova, A.; Mikhaylin, S.; Bazinet, L. Adsorption of Anthocyanins by Cation and Anion Exchange Resins with Aromatic and Aliphatic Polymer Matrices. *Int. J. Mol. Sci.* **2020**, *21*, 7874, doi:10.3390/ijms21217874.

Disclaimer/Publisher's Note: The statements, opinions and data contained in all publications are solely those of the individual author(s) and contributor(s) and not of MDPI and/or the editor(s). MDPI and/or the editor(s) disclaim responsibility for any injury to people or property resulting from any ideas, methods, instructions or products referred to in the content.
